# Developmental *Syngap1* Haploinsufficiency in Medial Ganglionic Eminence-Derived Interneurons Impairs Auditory Cortex Activity, Social Behavior, and Extinction of Fear Memory

**DOI:** 10.1523/JNEUROSCI.0946-24.2024

**Published:** 2024-10-15

**Authors:** Vidya Jadhav, Maria Isabel Carreno-Munoz, Pegah Chehrazi, Jacques L. Michaud, Bidisha Chattopadhyaya, Graziella Di Cristo

**Affiliations:** ^1^CHU Sainte-Justine Azrieli Research Centre (CHUSJ), Montréal, Quebec H3T 1C5, Canada; ^2^ Department of Neurosciences, Université de Montréal, Montréal, Quebec H3T 1J4, Canada; ^3^Department of Pediatrics, Université de Montréal, Montréal, Quebec H3T 1C5, Canada

**Keywords:** auditory cortex, fear memory extinction, GABAergic interneurons, parvalbumin cells, social behavior, Syngap1

## Abstract

Mutations in SYNGAP1, a protein enriched at glutamatergic synapses, cause intellectual disability associated with epilepsy, autism spectrum disorder, and sensory dysfunctions. Several studies showed that Syngap1 regulates the time course of forebrain glutamatergic synapse maturation; however, the developmental role of Syngap1 in inhibitory GABAergic neurons is less clear. GABAergic neurons can be classified into different subtypes based on their morphology, connectivity, and physiological properties. Whether *Syngap1* expression specifically in parvalbumin (PV)-expressing and somatostatin (SST)-expressing interneurons, which are derived from the medial ganglionic eminence (MGE), plays a role in the emergence of distinct brain functions remains largely unknown. We used genetic strategies to generate *Syngap1* haploinsufficiency in (1) prenatal interneurons derived from the medial ganglionic eminence, (2) in postnatal PV cells, and (3) in prenatal SST interneurons. We further performed in vivo recordings and behavioral assays to test whether and how these different genetic manipulations alter brain function and behavior in mice of either sex. Mice with prenatal-onset *Syngap1* haploinsufficiency restricted to Nkx2.1-expressing neurons show abnormal cortical oscillations and increased entrainment induced by 40 Hz auditory stimulation but lack stimulus-specific adaptation. This latter phenotype was reproduced in mice with *Syngap1* haploinsufficiency restricted to PV, but not SST, interneurons. Prenatal-onset *Syngap1* haploinsufficiency in Nkx2.1-expressing neurons led to impaired social behavior and inability to extinguish fear memories; however, neither postnatal PV- nor prenatal SST-specific mutant mice show these phenotypes. We speculate that *Syngap1* haploinsufficiency in prenatal/perinatal PV interneurons may contribute to cortical activity and cognitive alterations associated with *Syngap1* mutations.

## Significance Statement

Mutations in the human gene cause a form of developmental epileptic encephalopathy associated with intellectual disability, autism, and sensory dysfunctions. Several studies have shown that in addition to playing a major role in the synaptic maturation and plasticity of forebrain excitatory neurons, Syngap1 affects GABAergic circuit function as well. Forebrain GABAergic neurons can be divided into different subtypes. Whether *Syngap1* expression specifically in distinct interneuron populations and during specific developmental time windows plays a role in the emergence of distinct brain functions remains largely unknown. Here, we report that early, prenatal, or perinatal *Syngap1* expression in developing GABAergic neurons derived from the medial ganglionic eminence promotes the development of auditory cortex function, social behavior, and ability to extinguish fear memories.

## Introduction

*SYNGAP1* codes for a Ras GTPase-activating protein that is highly abundant in the postsynaptic density of excitatory synapses (Komiyama et al., 2002; Kim et al., 2003; Vazquez et al., 2004). Work spanning two decades led to the discovery of multiple functions of Syngap1, including the regulation of plasticity-associated signaling pathways, structural roles in regulating postsynaptic density composition at cortical glutamatergic synapses (Komiyama et al., 2002; Kim et al., 2003; Vazquez et al., 2004; Clement et al., 2012; Ozkan et al., 2014; Araki et al., 2015, 2020; Zeng et al., 2016), and, more recently, nonsynaptic function in cortical neurogenesis (Birtele et al., 2023). In children, *SYNGAP1* haploinsufficiency causes a developmental epileptic encephalopathy characterized by moderate to severe intellectual deficiency, generalized epilepsy (Hamdan et al., 2009; Rauch et al., 2012; Berryer et al., 2013; Carvill et al., 2013; von Stulpnagel et al., 2015; Mignot et al., 2016), and behavioral anomalies, including autism spectrum disorder and sensory processing impairment (Hamdan et al., 2011; Berryer et al., 2013; Michaelson et al., 2018; Carreno-Munoz et al., 2022; Lyons-Warren et al., 2022; Wright et al., 2022). Recent estimates suggest that *SYNGAP1*-ID may represent one of the most common monogenic forms of intellectual disability (Hamdan et al., 2009; Berryer et al., 2013, 2016; Parker et al., 2015), therefore making *SYNGAP1* haploinsufficiency one of the most frequent causes of genetically defined childhood brain disorders. How *Syngap1* regulates the development of different brain circuits and how its haploinsufficiency may contribute to specific phenotypes are still open questions and the focus of ongoing studies.

*Syngap1* haploinsufficiency is associated with premature excitatory synaptic maturation in the first postnatal weeks of mouse cortical development, which could in turn affect activity-dependent postnatal refinement of cortical circuits and alter long-range circuit connectivity (Clement et al., 2012, 2013; Aceti et al., 2015). Furthermore, *Syngap1* haploinsufficiency restricted to forebrain glutamatergic neurons is sufficient to recapitulate several of the phenotypes observed in *Syngap1^+/−^* mice, such as increased seizure threshold, hyperlocomotion, and deficits in working memory (Komiyama et al., 2002; Kim et al., 2003; Ozkan et al., 2014; Berryer et al., 2016; Creson et al., 2019; Nakajima et al., 2019). While *Syngap1* expression appears to be enriched in cortical and hippocampal glutamatergic neurons, it is also expressed in GABAergic neurons (Zhang et al., 1999; Berryer et al., 2016; Su et al., 2019; Zhao and Kwon, 2023; Allen Brain Institute 10x Genomics human tissue). Consistent with this observation, mounting evidence suggests that *Syngap1* affects the development and function of GABAergic inhibitory interneurons. For example, *Syngap1* was shown to be essential for the proper migration of inhibitory neurons to dorsal cortices during development (Su et al., 2019). Furthermore, early postnatal *Syngap1* deletion in sparse parvalbumin (PV)-expressing GABAergic cells in otherwise wild-type cortical organotypic cultures leads to a significant reduction of their perisomatic bouton density, suggesting a cell-autonomous role of *Syngap1* in the maturation of PV interneuron synaptic innervation (Berryer et al., 2016). Furthermore, mice where *Syngap1* haploinsufficiency is restricted to GABAergic interneurons, using the vesicular GABA transporter (Vgat) promoter to drive Cre expression, show impaired sensory learning in a whisker-guided tactile go/no-go task (Zhao and Kwon, 2023). Finally, we previously observed that mice where *Syngap1* haploinsufficiency is restricted to neurons derived from the medial ganglionic eminence (MGE), including PV- and SST-expressing interneurons, show behavioral deficits in the social domain (Berryer et al., 2016). Cortical GABAergic interneurons can be divided into different populations based on their gene expression profile, connectivity, and physiological properties, and this diversity likely contributes to different functional roles in cortical circuits (Kepecs and Fishell, 2014; Tremblay et al., 2016). Whether *Syngap1* differently affects different interneuron types is unknown. Furthermore, whether *Syngap1* function in GABAergic interneurons is limited to early development or extends to later stages is unclear. Here, we address these questions by using different conditional transgenic mouse strategies, in vivo recordings, and behavioral assays.

## Materials and Methods

### Mice

All procedures and experiments were done in accordance with the Comité Institutionnel de Bonnes Pratiques Animales en Recherche of the CHU Sainte-Justine Research Center in line with the principles published in the Canadian Council on Animal Care. *Syngap1^lox^* mice were purchased from the Jackson Laboratory (#029303). All animals were maintained under a light/dark cycle (12 h light/dark) in a temperature- and humidity-controlled room. Food and water were available *ad libitum*. *Syngap1^lox^* mice were crossed either with the Nkx2.1-Cre mice (Jackson Laboratory #008661), PV-Cre mice (Jackson Laboratory #008069), or SST-Cre (Jackson Laboratory #018973) mice. To quantify SST and PV interneuron density, we crossed these mice with mice carrying the RCE allele (Jackson Laboratory #032037). In the RCE allele, a STOP cassette flanked by the loxP sites was inserted upstream of the coding sequence of EGFP, thus allowing EGFP expression in Cre-expressing cells. The resulting genotypes were as follows: Tg(Nkx2.1-Cre);*RCE^lox/lox^;Syngap1^+/+^* (Nkx2.1-Cre control) and Tg(Nkx2.1-Cre);*RCE^lox/lox^;Syngap1^lox/+^* (Nkx2.1-Cre cHET). Tg(Nkx2.1-Cre);*RCE^lox/lox^;Syngap1^lox/+^* mice were further crossed together to obtain Tg(Nkx2.1-Cre);*RCE^lox/lox^;Syngap1^lox/lox^* mice (Nkx2.1-Cre cKO) along with control and Nkx2.1-Cre cHET littermates. We also generated the following genotypes: Tg(PV-Cre);*RCE^lox/lox^;Syngap1^+/+^* (PV-Cre control), Tg(PV-Cre);*RCE^lox/lox^;Syngap1^lox/+^* (PV-Cre cHET), Tg(SST-Cre);*RCE^lox/lox^;Syngap1^+/+^* (SST-Cre control), and Tg(SST-Cre);*RCE^lox/lox^;Syngap1^lox/+^* (SST-Cre cHET). Mice of both sexes were used for all experiments except for fear conditioning, which was performed using males only.

#### Genotyping

DNA was extracted from mouse tails using the AccuStart II Mouse Genotyping kit (#95135-500). Polymerase chain reaction (PCR) was done to detect the presence of Cre, RCE, *Syngap1^lox^*, and wild-type alleles. To detect the *Syngap1^lox^* alleles, two separate primers were used (F1 5′-GGGCTGTAAAACCCAACAAG-3′ and R1 5′-GCAGCTTTTTCTCAGGGAAC-3′) to detect a band size at 420 bp for the floxed allele and 372 bp for the WT allele. To detect the Nkx2.1-Cre transgene, three separate primers were used (F1 5′-AAGGCGGACTCGGTCCACTCCG-3′, F2 5′-TCCTCCAGGGGACTCAAGATG-3′, and R1 5′-TCGGATCCGCCGCATAACCAG-3′) to detect a band size at 220 bp for wild-type and 550 bp for the transgene. For PV-Cre transgene, three separate primers were used (F1 5′-CAGCCTCTGTTCCACATACACTCC-3′, F2 5′-GCTCAGAGCCTCCATTCCCT-3′, and R1 5′-TCACTCGAGAGTACCAAGCAGGCAGGAGATATC-3′) to detect a band size at 526 bp for wild-type and 400 bp for the transgenic allele. To detect the SST-Cre transgene, three separate primers were used (F1 5′-TCTGAAAGACTTGCGTTTGG-3′, F2 5′-TGGTTTGTCCAAACTCATCAA-3′, and R1 5′-GGGCCAGGAGTTAAGGAAGA-3′) to detect a band size at 465 bp for wild-type and 200 bp for the mutant allele. The RCE allele was detected using three separate primers (RCE-Rosa1 F1 5′-CCCAAAGTCGCTCTGAGTTGTTATC-3′, RCE-Rosa2 F2 5′-GAAGGAGCGGGAGAAATGGATATG-3′, and RCE-Cag3 R1 5′-CCAGGCGGGCCATTTACCGTAAG-3′) to detect band sizes at 550 and 350 bp for wild-type and mutant allele, respectively.

### Immunofluorescence analysis

P60 mice were anesthetized with [ketamine (100 mg/kg) plus xylazine (10 mg/kg) plus acepromazine (10 mg/kg)] and perfused transcardially with 0.9% saline followed by 4% paraformaldehyde (PFA) in phosphate buffer (0.1 M PB, pH 7.2–7.4). Brains were dissected out and postfixed in 4% PFA overnight at 4°C. They were subsequently transferred to 30% sucrose (prepared in PBS, pH 7.2) at 4°C for 48 h. Brains were then embedded in molds filled with OCT Tissue Tek and frozen in a bath of 2-methylbutane placed on a bed of dry ice and ethanol. Coronal sections were cut at 40 μm with a cryostat (Leica CM3050 S) and collected as floating sections in 1× PBS. Brain sections were blocked in 10% normal goat serum (NGS) with 1% Triton X-100 in PBS for 2 h at room temperature (RT) followed by incubation at 4°C for 48 h with the following primary antibodies diluted in 5% NGS and 0.1% Triton X-100 in PBS: rabbit anti-PV (1:5,000, Swant, catalog #PV27), rat anti-SST (1:500, Millipore, catalog #MAB354), mouse anti-NeuN (1:500, Millipore, catalog #MAB377), and chicken anti-GFP (1:500, Abcam, catalog #13970). Sections were then washed in PBS plus 0.1% Triton X-100 (3× 10 min each) and incubated for 2 h at RT with the following secondary antibodies diluted in 5% NGS and 0.1% Triton X-100 in PBS: Alexa 488-conjugated goat anti-chicken (1:1,000, Abcam, catalog #ab150169), Alexa 555-conjugated goat anti-rabbit (1:500, Life Technologies, catalog #A21430), Alexa 647-conjugated goat anti-mouse (1:500, Cell Signaling Technology, catalog #4410), Alexa 555-conjugated goat anti-rat (1:500, Life Technologies, catalog #A21434). Sections were rinsed in PBS (3× 10 min each) and mounted with Vectashield mounting medium (VectorLabs). Immunostained sections were imaged using a Leica SP8-DLS confocal microscope, with a 20× magnification (NA 0.75) at 1,024 × 1,024, zoom at 1, *z*-step of 1.5 μm, and stack size of ∼20 μm. Images were acquired from at least three coronal sections for each animal. Acquisition and analysis of images were done by an experimenter blind to the genotype. For neuron density quantification, 3–4 ROIs were drawn in each of the layers, and the number of cells was quantified using the cell counting option in Neurolucida (Microbrightfield Bioscience).

### Fluorescence multiplex RNAscope

Mice were cervically dislocated, and brains were dissected out at two ages, i.e., P20 and P60. Brains were washed with PBS and fast-frozen for ∼30–60 s in a cold bath of 2-methylbutane on a bed of dry ice and ethanol and preserved at −80°C. Twenty-micrometer coronal sections were cut with a cryostat (Leica Microsystems) and serially mounted (3–4 sections per slide) on Superfrost Plus Gold glass slides (Thermo Fisher Scientific catalog #12-550-15), which were then stored at −80°C until further use. RNAScope Multiplex Fluorescent Reagent Kit v2 for the reagents was purchased from Advanced Cell Diagnostics (catalog #320851) along with the following probes: Mm-Syngap1 - Mus musculus synaptic Ras GTPase-activating protein 1 homolog (rat) (Syngap1) for *Syngap1* mRNA (catalog #417381), Mm-Pvalb-C2 - Mus musculus parvalbumin (Pvalb) mRNA for *Pvalb* mRNA (catalog #421931-C2), Mm-Sst-C2 - Mus musculus somatostatin (Sst) for *Sst* mRNA (catalog #404631-C2), positive probe (catalog #320881), and negative probe (catalog #320871). The entire procedure for tissue pretreatment, hybridization, amplification, and detection was performed as per the RNAscope Multiplex Fluorescent Assay (Advanced Cell Diagnostics) manual for fresh frozen tissue. For pretreatment, slides were removed from −80°C and immediately postfixed in 4% PFA at 4°C for 15 min followed by dehydration in 50, 70, and 2× 100% ethanol for 5 min, respectively, at room temperature (RT). The slides were air-dried, and a hydrophobic barrier was created around the section with an ImmEdge Pen (H-4000). Slides were treated with protease IV for 15 and 30 min, for P20 and P60 brain sections respectively, at RT, and then washed twice in PBS. All the probes were prewarmed at 40°C in a water bath for 10 min and cooled at RT. For hybridization, the probes were incubated at 40°C for 2 h. Amplification steps were carried out as per the protocol. For detection of the probes, Amp4B was used (C1 probes-Atto 550 and C2 probes-Alexa-488). Sections were stained with DAPI (320858) for 30 s and mounted with ProLong Diamond Antifade (Thermo Fisher Scientific P36961). Images of the primary cortical auditory cortex were taken with an SP8-STED confocal microscope using a 63× magnification (NA 1.4) at 2,048 × 2,048, zoom of 2, and *z*-step of 0.3 μm with gating between 0.3 and 6.0 ms. At least three brain slices/mouse were imaged. Images were deconvolved using Huygens HyVolution developed for Leica Systems. Quantification of mRNA was performed using Fiji (ImageJ), as described by Lavertu-Jolin et al. (2023). Putative cell somata were manually selected by using DAPI staining and either *Pvalb* or *Sst* puncta distribution. Cells were duplicated, separated into channels, and filtered using 3D Gaussian blur. Dots were quantified by using the 3D object counter in ImageJ Fiji, after setting an appropriate threshold value for each of the probes. Brain slices from P20 and P60 mice were processed at the same time. The results were reported as the mean number of dots per cell.

### Behavioral analysis

Mice of either sex, unless differently specified, were housed under a 12 h light/dark cycle in a temperature- and humidity-controlled room. Food and water were available *ad libitum*. Mice were habituated to the operator prior to all behavior experiments. All behavior experiments were performed with the light intensity range of 60–100 lux. A camera was mounted above the arena for video recording during the behavioral task and controlled by SMART video tracking software (Panlab v3.0, Harvard Apparatus) or Freeze Frame software IMAQ 3 (Version 3.0.1.0). The sequence of tested mice was randomized by the genotype. Mice were tested between P60 and P90. Each behavioral apparatus was cleaned with 70% ethanol between each trial. Experimenters were blind to genotypes during both testing and data analysis. All behavioral experiments were done using at least 3–4 cohorts from different mouse litters. If not differently specified, we use both male and female mice for these studies.

#### Open field

Each mouse was gently placed in a corner of the open field apparatus (box dimension, 45 × 45 cm) and allowed to explore for 10 min. No mice were excluded from the analysis.

#### Elevated plus maze

The apparatus consists of two open arms without walls facing across each other and perpendicular to two closed arms with walls joining at the central platform. Each mouse was placed at the center of the junction of two open and closed arms and allowed to explore for 5 min. No mice were excluded from the analysis.

#### Novel object recognition

Each mouse was habituated to the open field box for a duration of 5 min. For the familiar phase, 1 h after habituation, two familiar objects (two similar cylindrical objects: white in color) were placed in the box at one-third the distance from one end of the box. Each mouse was placed at one end of the box facing the wall away from the objects and allowed to explore for 10 min. Only mice that explored the objects for at least 10 s were considered for further analysis. Based on this criterion, we excluded two Nkx2.1-Cre cHET and two Nkx2.1-Cre Ctrl mice. The discrimination index (DI) was calculated as (Time spent with Object 1 − Time spent with Object 2) / (Total time spent with Object 1 + Object 2). Two hours later, one of the familiar objects was replaced with a novel object (cone-shaped object; white in color). Each mouse was allowed to explore for 10 min, and DI was calculated as (Time spent with novel object − Time spent with familiar object) / (Total time spent with novel object + familiar object). The placement of the familiar and the novel objects was randomized for each mouse.

#### Three-chamber test

Each mouse was placed in the center of a three-chamber rectangular box (dimension, 61 × 42.5 cm) and allowed to explore for 10 min (habituation). Next, a wire cage with an unfamiliar conspecific mouse of the same size, age, and sex (Stranger 1) was placed on one side of the chamber, while an empty wire cage was placed on the other side of the chamber. The test mouse was placed in the center of the chamber and allowed to explore for 10 min (social approach). Finally, a novel unfamiliar mouse (Stranger 2) of the same size, age, and sex was placed in the previously unoccupied empty cage, and the test mouse was allowed to explore for 10 min (social novelty). Strangers 1 and 2 were taken from different home cages and have never been in close physical contact with the test mouse or with each other. No mice were excluded from the analysis.

#### Contextual fear conditioning

Male mice were habituated on Day 1 and allowed to freely explore for 3 min in the test context, which consisted of white walls and a grid floor for habituation. On Day 2, mice were fear conditioned using three random unconditioned stimuli (US; 2 s footshock, 0.5 mA), delivered over a period of 5 min. Mice were tested in the same context both 24 h and 30 d later. An experimenter blind to the genotype scored freezing behavior defined by the time the mouse remained in complete immobility with the exception of respiratory movements, during the 5 min period spent in the context. No mice were excluded from the analysis.

#### Cued fear conditioning and fear extinction

Fear acquisition and extinction experiments were performed as described by Lavertu-Jolin et al. (2023), using two different contexts (Context A and Context B, respectively). Context A consisted of white walls and a grid floor and was cleaned with 70% ethanol before and after each session. Context B consisted of walls with black and white stripes and a white plexiglass floor and was cleaned with 1% acetic acid before and after each session. On Day 1, mice were allowed to freely explore for 3 min in Context A (habituation). On Day 2, mice were conditioned with five random pairings of the conditioned stimulus (CS; duration, 5 s; white noise, 80 dB) with coterminating US (2 s footshock, 0.6 mA; intertrial interval, 30–60 s). On Days 3 and 4, mice were subjected to early and late extinction training in Context B with 12 presentations of CS at 30 s intervals on each day. Fear retrieval and renewal were tested 7 d later in Contexts B and A, respectively, using four CS presentations at an interval of 30 s. Mouse behavior was video-recorded with Freeze Frame software. An experimenter blind to the genotype scored freezing behavior (defined by complete immobility with the exception of respiratory movements) by measuring time spent freezing for 30 s following CS presentation for extinction and during 1 min following CS presentation in retrieval and renewal. The results presented are the mean of time spent freezing after the presentation of the first two CS on the first day of extinction (Day 2; CS1–2, early extinction), the last two CS on the second day of extinction (Day 3; CS 11–12, late extinction), the first two CS during the retrieval test in the extinction Context B (Day 10; CS 1–2, retrieval), and the first two CS during the renewal test in the fear acquisition Context A (Day 10; CS 1–2, renewal). No mice were excluded from the analysis.

### In vivo electrophysiology

#### Surgery

For EEG recordings in anesthetized mice, anesthesia was induced by an intraperitoneal injection of urethane using an initial large dose (1.5 g/kg, i.p.) followed by supplementary small doses (0.1–0.15 g/kg) during the recording, if necessary, as indicated by the presence of a withdrawal response of the limb to pinch. The animal was transferred to the stereotaxic apparatus for both surgery and subsequent recording. A craniotomy was performed on the right primary auditory cortex of the brain at the following coordinates: −2.5 mm posterior to bregma, 3.9 mm lateral to the midline, and 0.8 mm deep. A second craniotomy was performed in the contralateral prefrontal lobe where the reference and ground reference wire were placed. Custom-made electrodes were made with a cluster of five 50-µm-diameter insulated tungsten wires. Wire tips were 100 µm apart to reach different depths. Body temperature was maintained at 36–37°C by a thermostatically controlled heating pad.

For EEG recordings in awake mice, electrodes were implanted under deep anesthesia, induced by an intraperitoneal injection of ketamine/xylazine/acepromazine cocktail (80 mg/kg ketamine, 15 mg/kg xylazine, and 2 mg/kg acepromazine). A craniotomy was performed on the right side of the primary auditory cortex as described for recording in anesthetized mice. Custom-made clusters of four to six 50-μm-diameter insulated tungsten wires were implanted. Wire tips (recording sites) were 50–150 μm apart. A ground-and-reference wire was also gently introduced in the contralateral frontal lobe, and a 2 cm carbon fiber bar was placed on the back of the skull. The whole structure was then daubed with dental acrylic to encase the electrode–microdrive assembly and anchor it to the skull. During the whole surgery, body temperature was maintained at 36–37°C by a thermostatically controlled heating pad. For a week after surgery, animals were treated with Metacam, and the skin around the implant was disinfected daily for 3 d following the surgery. Experimenters were blind during surgery, data acquisition, and analysis.

#### EEG recording

EEG recordings were performed using an Open Ephys GUI platform (https://open-ephys.org/) at a sampling rate of 20 kHz. Data were acquired with a custom-made headstage using Intan Technologies’ RHD Electrophysiology Amplifier Chip. EEG data and video were recorded simultaneously. Custom-designed stimulus trigger generator devices sent triggers directly to the recording system assuring the time precision of the stimulus presentation.

#### Sensory stimulations and analysis

These experiments were performed as described by Carreno-Munoz et al. (2022). All stimulations were presented at an intensity of 70 dB. For pure tone stimulation, we used 100-ms-long 5 and 10 kHz pure tones presented with a random interstimulus interval of 2–3 s. Each pure tone was presented 60 times in random order. The full protocol lasted 5 min. For the habituation/oddball paradigm, we used sequences of 5–9 repetitive (standard, at 5 kHz) sounds followed by a deviant sound (at 10 kHz). Sequences were presented in a pre-established pseudorandom order. Sounds lasted 70 ms and were presented at 1 s interstimulus interval. The full protocol lasted 10 min. For auditory steady-state stimulation, we used 10 and 40 Hz click trains (1 s duration and 2.5 s intertrial interval), presented at alternating frequencies. Each click was a sound at 5 kHz, lasting 5 ms. This protocol lasted 5 min.

#### EEG preprocessing

The mouse EEG signals were downsampled to 2,000 Hz and filtered with a passband filter (0.5–150 Hz) and a notch filter (59.5–60.5 Hz) to remove residual 60 Hz powerline noise contamination. Data were then segmented into periods of different lengths, depending on the stimulation protocol: 1,400 ms (500 ms pre- and 900 ms poststimulus onset) for the auditory oddball protocol; 2,500 ms (1,000 ms pre- and 1,500 ms poststimulus onset) for the pure tone stimulation; and 4,000 ms (1,000 ms pre- and 3,000 ms poststimulus onset) for auditory steady state. Trials containing per-sample segments with an intrachannel average >4 times the total trial standard deviation were tagged, visually inspected, and removed. Only sessions containing >50 clean trials were kept for further analyses. Signal analysis and quantification was performed using custom MATLAB (MathWorks) code, available upon request.

#### Auditory-evoked potentials

The EEG signal was low-pass filtered to 150 Hz, baseline corrected to the mean voltage of the 150 ms prior to stimulus onset, and averaged over the trial. Event-related potential components baseline-to-peak N1 and P1 were analyzed from the AEP. The N1 amplitude was automatically detected by subtracting the minimum voltage (negative peak) within a 10–80 ms time window after stimulus onset to the averaged baseline value. The P1 amplitude was extracted by subtracting the maximum voltage (positive peak) within an 80–150 ms time window after stimulus onset to the averaged baseline value. Analyses were carried out using FieldTrip toolbox v. 202009.

#### Habituation and mismatch negativity

To quantify habituation in mice, we calculated the ratio between the amplitudes of the first standard sound (S1) and the second (S2). These ratios were then converted to a logarithmic base 10 scale. Mismatch negativity (MMN) was calculated by subtracting the EEG trace of the standard sound from the EEG trace of the deviant sound. The pattern obtained was then used to detect N1 and P1 amplitudes in each subject.

Intertrial coherence. Intertrial coherence allows assessment of the strength of phase coherence across trials in temporal and spectral domains. The intertrial coherence computation uses only the phase of the complex values given by Morlet's wavelet transform. Intertrial coherence measures phase coupling across trials at all latencies and frequencies and is defined as follows:

$$\mathrm{IT}C_{t}=\frac{1}{N}\sum_{n=1}^{N}\exp\;(\;j\Theta (\;f,t,n)),$$

where jΘ(f,t,n) represents the phase for a given frequency (*f*), time point (*t*), and trial (*n*). The obtained values are always defined between 0 and 1. Phase-locking values close to 1 indicate strong intertrial phase-locking, thus representing evoked activity while scores closer to 0 indicate a high intertrial phase variability.

### Experimental design and statistical analysis

For each experiment, littermates of both genotypes, from at least three different litters, were analyzed. Data were first tested for normal distribution with D’Agostino and Pearson's test, Anderson–Darling test or Shapiro–Wilk test, and Kolmogorov–Smirnov test. Data involving only two experimental groups were analyzed using either (1) *t* test with Welch's correction for normally distributed data or (2) Mann–Whitney test for non-normally distributed data. Data with >2 experimental groups were analyzed using one-way ANOVA with post hoc Dunn's multiple-comparisons test for normally distributed data or Kruskal–Wallis test for non-normally distributed data. For the three-chamber test (social behavior), sniffing times were compared using two-way ANOVA with post hoc Holm–Sidak's multiple-comparisons test.

## Results

### *Syngap1* mRNA is expressed at higher levels in preadolescent than in adult PV^+^ and SST^+^ cortical interneurons

**Figure 1.**
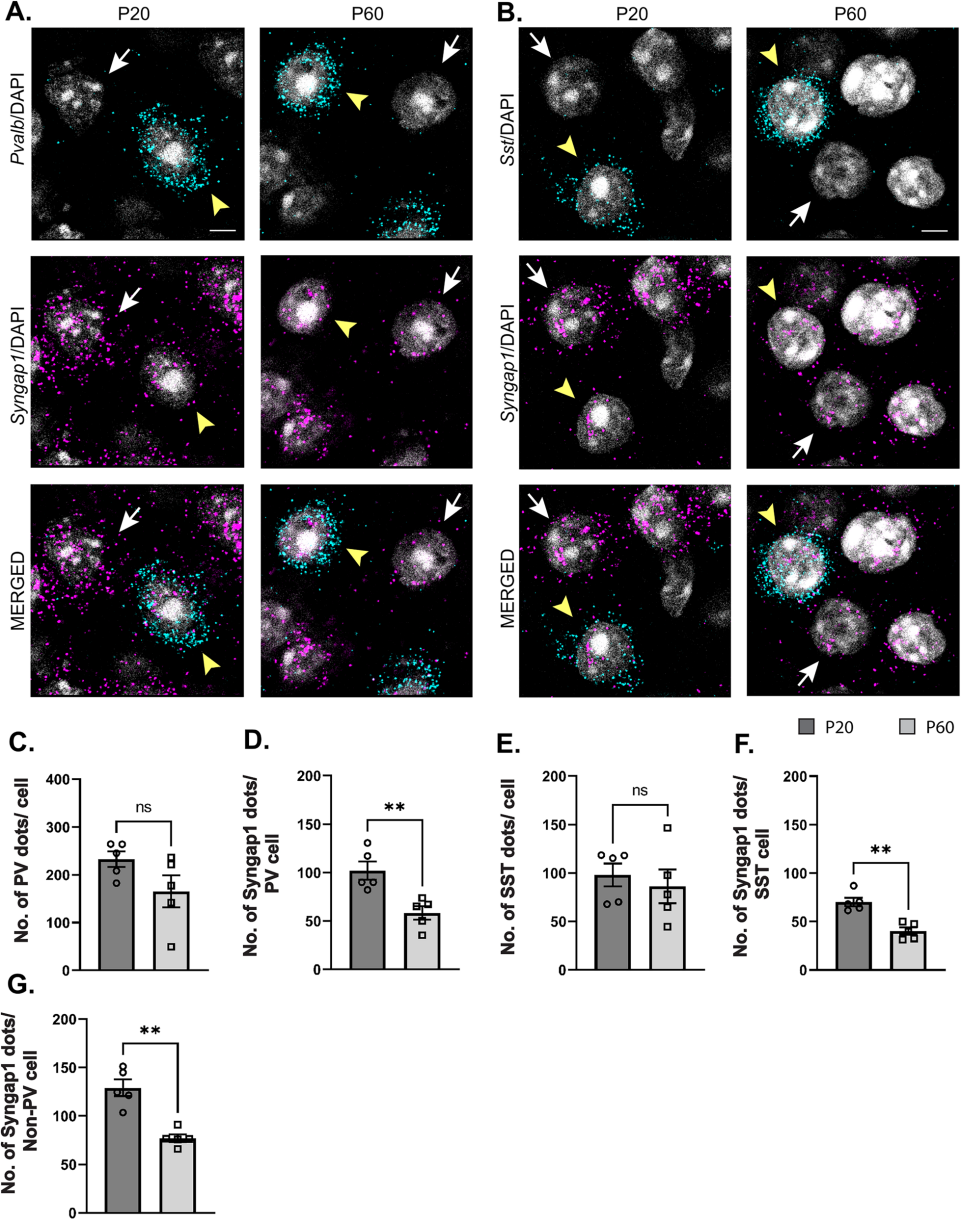
Syngap1 transcripts are significantly reduced in PV, SST, and non-PV cells in adult compared with preadolescent mice. ***A***, ***B***, Representative RNAscope images of layer 5 auditory cortex from P20 and P60 mice probed with *Pvalb* (cyan) and *Syngap1* (magenta; ***A***) or *Sst* (cyan) and *Syngap1* (magenta; ***B***). Cell nuclei are labeled with DAPI (gray). *Pvalb-*positive (***A***) and *Sst*-positive (***B***) cell somata are indicated by yellow arrowheads, while *Pvalb*-negative (***A***) or *Sst*-negative somata (***B***) are indicated by white arrows. Scale bar, 5 µm. ***C***, *Pvalb* transcript number per cell at P20 and P60 (Welch's *t* test, *p* = 0.1204). ***D***, *Syngap1* transcripts in *Pvalb*^+^ cells at P20 and P60 (Welch's *t* test, **p = 0.0064). ***E***, *Sst* transcript number per cell at P20 and P60 (Mann–Whitney test, *p* = 0.5476). ***F***, *Syngap1* transcripts in *Sst*^+^ cells at P20 and P60 (Welch's *t* test, **p = 0.0010). ***G***, *Syngap1* transcripts in *Pvalb*^−^ cells at P20 and P60 (Welch's *t* test, **p = 0.0018). Number of mice, *n* = 5 for both ages. Bar graphs represent mean ± SEM. *p* > 0.05, not significant (ns), *p < 0.05, **p < 0.01, ***p < 0.001.

Syngap1 expression has been detected in a subset of GABAergic neurons in dissociated hippocampal and cortical neuronal cultures (Zhang et al., 1999; Moon et al., 2008; Berryer et al., 2016). Previous analysis of *Syngap1* expression in cortical interneuron subtypes using transcriptome datasets collected by the Allen Brain Institute suggests that *Syngap1* is detected in GABAergic interneurons and excitatory cells in the adult cortex (Zhao and Kwon, 2023). We first sought to confirm *Syngap1* expression in cortical PV and SST neurons and then investigated whether its expression may be different in the developing versus the adult cortex. Since Syngap1 immunostaining in mouse brain slices gives a diffuse signal with poor spatial resolution, we turned to RNAscope Multiplex Fluorescent in situ hybridization (Advanced Cell Diagnostics), which allows single-mRNA molecule detection. We simultaneously labeled the mRNA coding for SYNGAP1 (gene name, *Syngap1*) and either PV (gene name, *Pvalb*) or Somatostatin (gene name, *Sst*) in the cortex of preadolescent (P20) and young adult (P60) mice (Fig. 1*A,B*). We chose the two developmental points since cortical GABAergic connectivity undergoes a prolonged postnatal maturation phase, accelerating after the first postnatal week and reaching a plateau by the first postnatal month (Micheva and Beaulieu, 1996; Chattopadhyaya et al., 2004; Pangratz-Fuehrer and Hestrin, 2011; Favuzzi et al., 2019). We found that *Pvalb*- and *Sst*-positive interneurons expressed *Syngap1* at both ages. However, *Syngap1* mRNA particle numbers were significantly higher at P20 than at P60 in both GABAergic cell populations (Fig. 1*D,F*). This was not due to age-dependent changes in mRNA detection, since both *Pvalb* and *Sst* mRNA particle numbers were not significantly different at P20 versus P60 (Fig. 1*C,E*). To investigate whether the decrease in *Syngap1* expression was specific to GABAergic cells or a widespread phenomenon, we quantified *Syngap1* mRNA in *Pvalb*-negative neurons, which, most likely, represented pyramidal neurons, and found a similar age-dependent decrease (Fig. 1*G*). Of note, *Syngap1* mRNA particle numbers were significantly higher in *Pvalb*- than in *Sst*-positive interneurons at P20, but the difference did not reach significance at P60 (P20, Welch's *t* test, *p* = 0.0227; P60, Welch's *t* test, *p* = 0.0597; number of mice, *n* = 5 for both ages). When comparing *Syngap1* mRNA particle numbers *Pvalb*-positive versus *Pvalb*-negative (most likely excitatory neurons), we found that the latter showed a trend toward higher *Syngap1* expression (P20, Welch's *t* test, *p* = 0.0648; P60, Welch's *t* test, *p* = 0.0544; number of mice, *n* = 5 for both ages). These data suggest that PV neurons express higher levels of *Syngap1* mRNA; however, whether different mRNA particle number translates in different protein expression levels remains to be investigated.

### *Syngap1* haploinsufficiency in MGE-derived Nkx2.1-expressing interneurons does not alter interneuronal density or final positioning in the adult cortex

To investigate the role of *Syngap1* in the early development of PV and SST interneurons, we used *Nkx2.1Cre;RCE;Syngap1^lox/+^* mice to remove either one or both *Syngap1* alleles starting at around embryonic day (E) 10.5. Nkx2.1, a transcription factor expressed in the medial ganglionic eminence (MGE), is essential for the specification of cortical PV and SST interneurons (Q. Xu et al., 2004, 2008), while RCE is a reporter line expressing eGFP in a Cre-dependent manner. In this mouse line, we confirmed that the proportion of PV and SST neurons expressing GFP varied depending on the cortical layer and brain region. In particular, the proportion of PV cells expressing GFP was 66 ± 2% and 84 ± 2% in sensory cortex L23 and L56, respectively, and 88 ± 3% in the prefrontal cortex of adult mice (*N* = 6 mice). The proportion of SST cells expressing GFP was 57 ± 4% and 72 ± 9% in sensory cortex L23 and L56, respectively, and 73 ± 5% in the prefrontal cortex of adult mice (*N* = 3 mice). Not all cortical SST and PV interneurons express Cre most likely because the *Nkx2.1Cre* transgenic mouse was generated not by knock-in of Cre downstream the endogenous Nkx2.1 promoter but by genomic integration of a modified bacterial artificial chromosome, in which the second exon of Nkx2.1 was replaced by Cre (Q. Xu et al., 2008).

**Figure 2.**
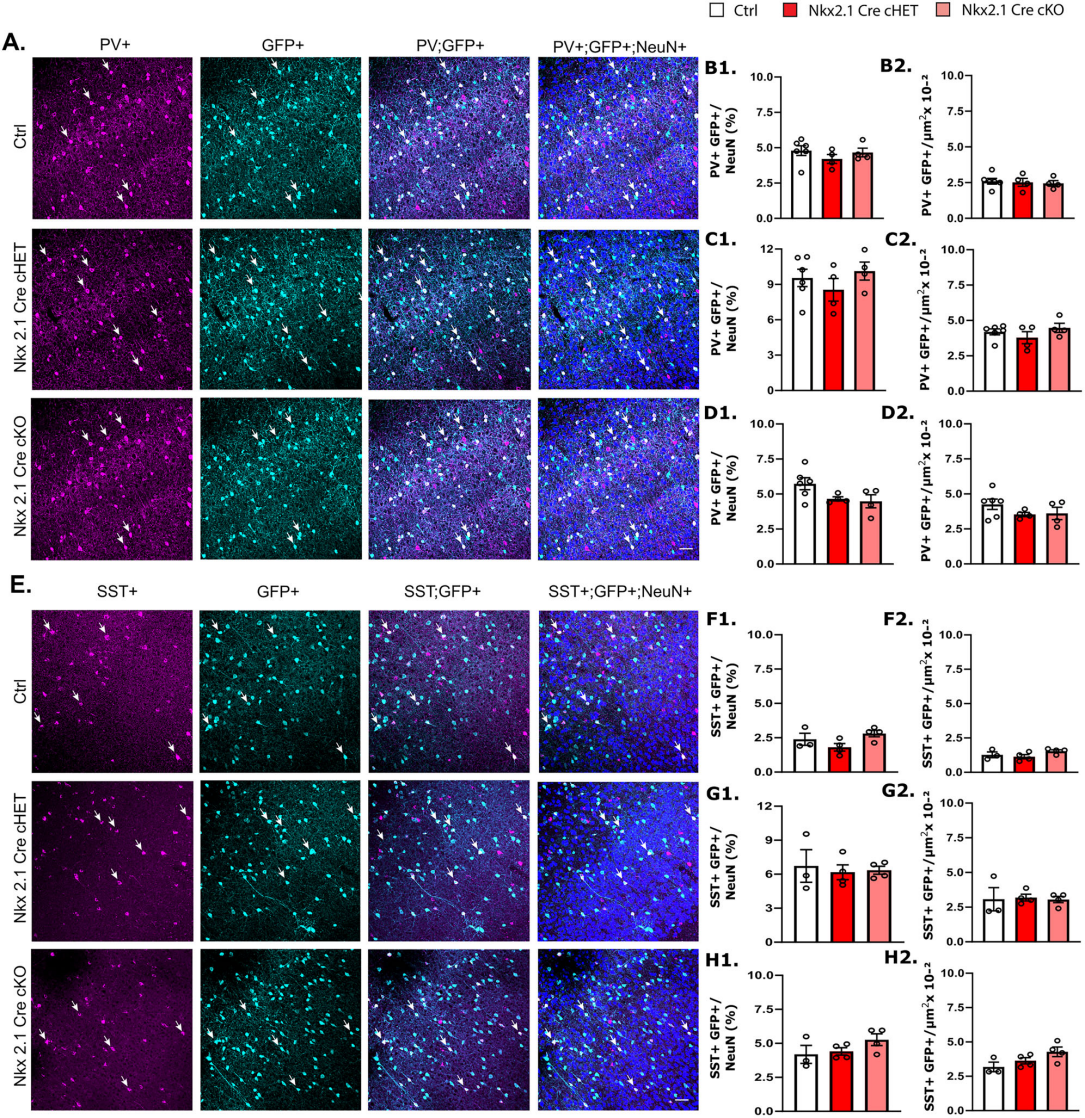
Density and distribution of PV- and SST-positive cells are not affected by haploinsufficiency or deletion of *Syngap1* in MGE-derived interneurons. ***A***, Representative image of cortical coronal sections from control and MGE-derived conditional mutant mice (heterozygous, Nkx2.1-Cre cHET; homozygous, Nkx2.1 Cre cKO) immunostained for PV (magenta), GFP (cyan), and NeuN (blue). Scale bar, 50 µm. ***B*1*–D*1**, Percentage of PV^+^ GFP^+^ over NeuN^+^ cells in sensory cortex layer 2/3 (***B*1**) and layer 5/6 (***C*1**) and in the prefrontal cortex (***D*1**). ***B*1**, One-way ANOVA with Dunnett's multiple-comparisons post hoc method, *p* = 0.3862 for Nkx2.1Cre-cHET and *p* = 0.9450 for Nkx2.1Cre-cKO versus Ctrl. ***C*1**, One-way ANOVA with Dunnett's multiple-comparisons post hoc method, *p* = 0.6095 for Nkx2.1Cre-cHET and *p* = 0.8389 for Nkx2.1Cre-cKO versus Ctrl. ***D*1**, One-way ANOVA with Dunnett's multiple-comparisons post hoc method, *p* = 0.1456 for Nkx2.1Cre-cHET and *p* = 0.0857 for Nkx2.1Cre-cKO versus Ctrl. ***B*2*–D*2**, Density of PV^+^ GFP^+^ per square micrometer in sensory cortex layer 2/3 (***B*2**) and layer 5/6 (***C*2**) and in the prefrontal cortex (***D*2**). ***B*2**, One-way ANOVA with Dunnett's multiple-comparisons post hoc method, *p* = 0.9546 for Nkx2.1Cre-cHET and *p* = 0.8474 for Nkx2.1Cre-cKO versus Ctrl. ***C*2**, One-way ANOVA with Dunnett's multiple-comparisons post hoc method, *p* = 0.5588 for Nkx2.1Cre-cHET and *p* = 0.7473 for Nkx2.1Cre-cKO versus Ctrl. ***D*2**, One-way ANOVA with Dunnett's multiple-comparisons post hoc method, *p* = 0.3033 for Nkx2.1Cre-cHET and *p* = 0.3698 for Nkx2.1Cre-cKO versus Ctrl. Number of mice, *n* = 6 Ctrl; *n* = 4 Nkx2.1Cre-cHET and *n* = 4 Nkx2.1Cre-cKO. ***E***, Representative image of cortical coronal sections from control and MGE-derived conditional mutants of adult mice immunostained for SST (magenta), GFP (cyan), and NeuN (blue). Scale bar, 50 µm. ***F*1*–H*1**, Percentage of SST^+^ GFP^+^ normalized over NeuN^+^ cells in sensory cortex layer 2/3 (***F*1**) and layer 5/6 (***G*1**) and in the prefrontal cortex (***H*1**). ***F*1**, One-way ANOVA with Dunnett's multiple-comparisons post hoc method, *p* = 0.3570 for Nkx2.1Cre-cHET and *p* = 0.5603 for Nkx2.1Cre-cKO versus Ctrl. ***G*1**, One-way ANOVA with Dunnett's multiple-comparisons post hoc method, *p* = 0.8491 for Nkx2.1Cre-cHET and *p* = 0.9244 for Nkx2.1Cre-cKO versus Ctrl. ***H*1**, One-way ANOVA with Dunnett's multiple-comparisons post hoc method, *p* = 0.9136 for Nkx2.1Cre-cHET and *p* = 0.2171 for Nkx2.1Cre-cKO versus Ctrl. ***F*2*–H*2**. Density of SST^+^ GFP^+^ per square micrometer in sensory cortex layer 2/3 (***F*2**) and layer 5/6 (***G*2**) and in the prefrontal cortex (***H*2**). ***F*2**, One-way ANOVA with Dunnett's multiple-comparisons post hoc method, *p* = 0.7498 for Nkx2.1Cre-cHET and *p* = 0.4087 for Nkx2.1Cre-cKO versus Ctrl. ***G*2**, One-way ANOVA with Dunnett's multiple-comparisons post hoc method, *p* = 0.9713 for Nkx2.1Cre-cHET and *p* = 0.9992 for Nkx2.1Cre-cKO versus Ctrl. ***H*2**, One-way ANOVA with Dunnett's multiple-comparisons post hoc method, *p* = 0.5287 for Nkx2.1Cre-cHET and *p* = 0.0697 for Nkx2.1Cre-cKO versus Ctrl. Number of mice, *n* = 3 Ctrl; *n* = 4 Nkx2.1Cre-cHET and *n* = 4 Nkx2.1Cre-cKO. Bar graphs represent mean ± SEM. *p* > 0.05, not significant (ns), *p < 0.05, **p < 0.01, ***p < 0.001.

*Syngap1* has been shown to be involved in the migration of inhibitory neurons during development (Su et al., 2019); therefore, conditional prenatal deletion of *Syngap1* may affect their final numbers in the adult cortex by altering their final positioning. Here, we used the *Syngap1^lox^* transgenic line reported previously (Clement et al., 2012; Ozkan et al., 2014) wherein the loxP sites flank Exons 6–7 (hereafter indicated simply as *Syngap1^lox^*). Neuron-type–specific Cre recombinase expression in this mouse line led to the efficient removal of Syngap1 protein in both glutamatergic and GABAergic cortical cells (Ozkan et al., 2014). Neither conditional heterozygous (*Nkx2.1Cre;RCE;Syngap1^lox/+^* referred to hereafter as cHET) nor conditional homozygous (*Nkx2.1Cre; RCE;Syngap1^lox/lox^* referred to hereafter as cKO) mice showed significant difference in PV (Fig. 2*A,B*1–*D*1*,B*2*–D*2) and SST (Fig. 2*E,F*1*–H*1*,F*2*–H*2) neuron density compared with wild-type littermates (*Nkx2.1Cre; RCE;Syngap1^+/+^* referred to hereafter as control) in sensory (Fig. 2*B*1*–C*2*,F*1*–G*2) and associative cortices (Fig. 2*D*1*,D*2*,H*1*,H*2). Therefore, *Syngap1* deletion in Nkx2.1-expressing GABAergic progenitors does not appear to affect the survival and final positioning of adult cortical PV and SST interneurons.

### *Syngap1* haploinsufficiency restricted to Nkx2.1-expressing neurons leads to increased baseline gamma power in adult mice

**Figure 3.**
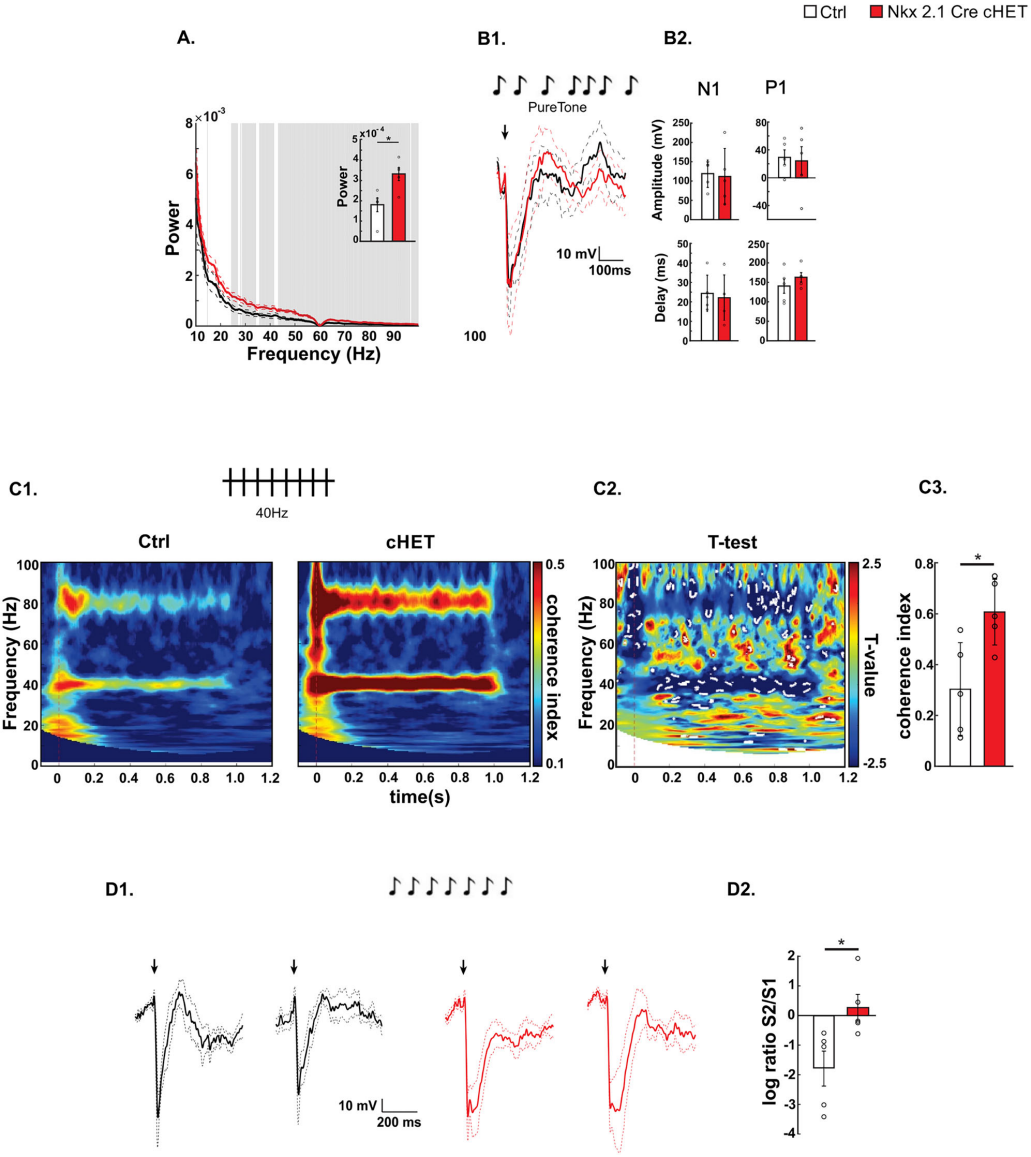
MGE-specific *Syngap1* haploinsufficiency leads to increased baseline gamma power and auditory entrainment at 40 Hz as well as decreased auditory habituation. ***A***, Power density spectra of 10 min baseline state (nonstimulus) EEG signal from ***A*1**. Gray bars represent significant differences (*t* test, *p* < 0.05). The appended bar plot shows mean powers ± SEM of the broadband gamma (30–100 Hz) oscillations (*t* test; *t*_(8)_ = −3.1670, *p* = 0.0133). ***B*1**, ***B*2**, AEP profile. ***B*1**, Superposed grand average traces. The black arrow represents the beginning of the stimulation. ***B*2**, Bar plots showing N1 (left) amplitude (top panel, Wilcoxon rank sum test; *z* = 0.6267, *p* = 0.5309) and delay (bottom panel, Wilcoxon rank sum test; *z* = −0.4191, *p* = 0.6752) and P1 (right) amplitude (top panel, Wilcoxon rank sum test; *z* = 0, *p* = 1) and delay (bottom panel, *t* test; *t*_(8)_ = −1.0182, *p* = 0.3384). ***C*1–*C*3**, Auditory entrainment at 40 Hz. ***C*1**, Intertrial coherence in control (left) and Tg(Nkx2.1-Cre);Syngap1^llox/+^ (right). ***C*2**, *t* test maps. Statistical differences (*p* < 0.05) are marked by white dotted lines. ***C*3**, Bar plots show coherence index values corresponding to the stimulating frequency, 40 Hz (*t* test, *t*_(8)_ = −3.0734, *p* = 0.0153). ***D*1**, AEP response to the first (left) and second (right) standard sound of control (black) versus Tg(Nkx2.1-Cre);Syngap1^llox/+^ (red) mice. ***D*2**, Bar plot shows logarithmic ratio S2/S1 (*t* test; *t*_(8)_ = −2.7333, *p* = 0.0257). Number of mice, *n* = 5 Ctrl; *n* = 5 Nkx2.1Cre-cHET. Bar graphs represent mean ± SEM. *p < 0.05.

**Figure 4.**
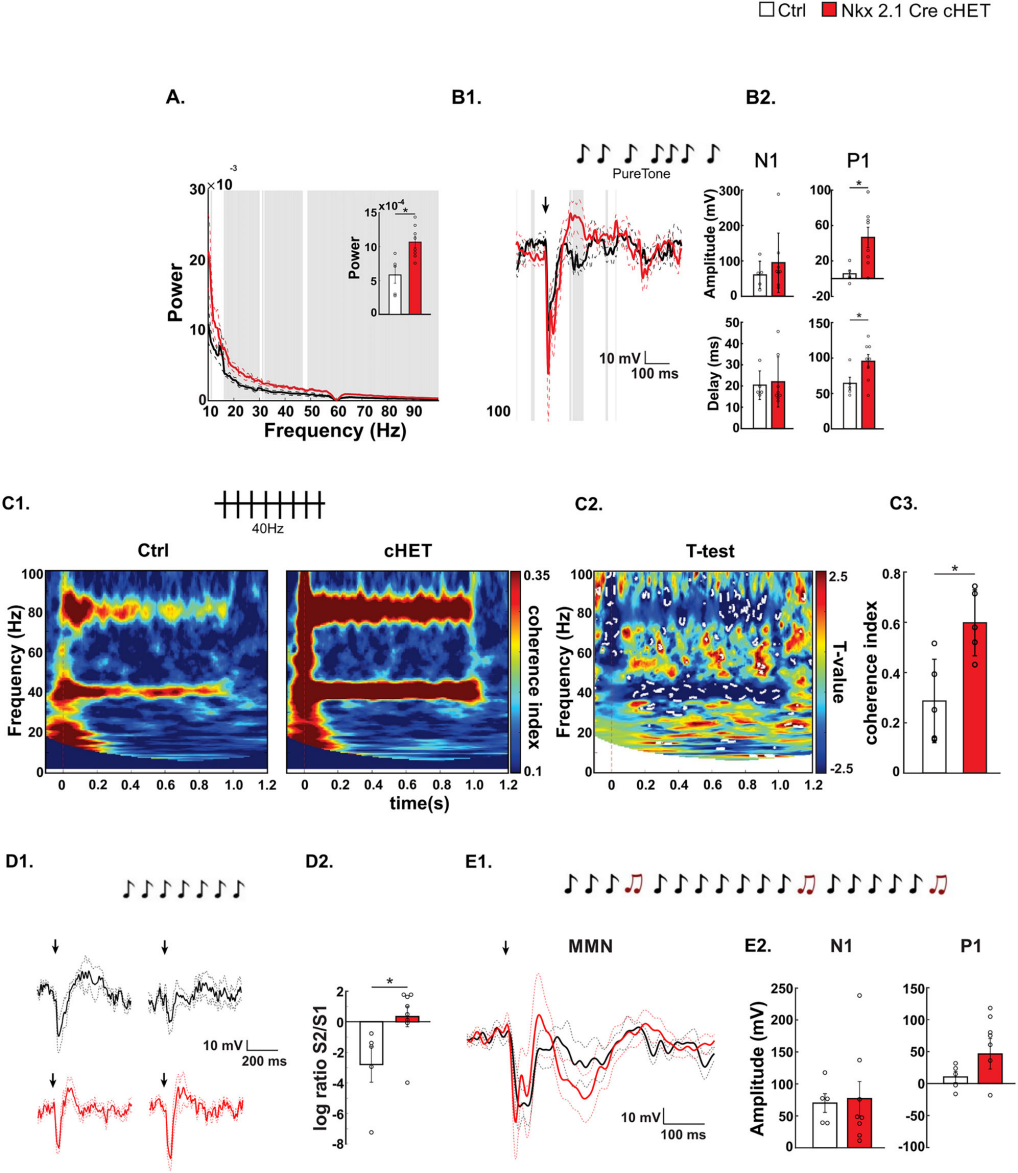
Awake MGE-restricted *Syngap1* haploinsufficient mice show increased baseline gamma power and auditory entrainment at 40 Hz as well as decreased auditory habituation. ***A***, Power density spectra of 3 min baseline state (nonstimulus) EEG signal. Gray indicates significant differences. The appended bar plot shows the mean power ± SEM of the broadband gamma (30–100 Hz) oscillations (Wilcoxon rank sum test; *z* = −2.5617, *p* = 0.0104). ***B*1**, ***B*2**, AEP profile. ***B*1**, Superposed grand average traces. The black arrow represents the beginning of the stimulation. ***B*2**, Bar plots showing N1 (left) amplitude (top panel, *t* test; *t*_(11)_ = −0.2860, *p* = 0.7802) and delay (bottom panel, *t* test; *t*_(11)_ = 0.8597, *p* = 0.4083) and P1 (right) amplitude (top, Wilcoxon rank sum test; *z* = −2.1226, *p* = 0.0338) and delay (bottom panel, *t* test; *t*_(11)_ = −2.1526, *p* = 0.0544). ***C*1–*C*3**, Auditory entrainment at 40 Hz. ***C*1**, Intertrial coherence in *control* (left) and *Tg(Nkx2.1-Cre);Syngap1^llox/+^* (right) mice. ***C*2**, *t* test maps. Statistical differences (*p* < 0.05) are marked by white dotted lines. ***C*3**, Bar plots show coherence index values at 40 Hz (*t* test, *t*_(8)_ = −3.0734, *p* = 0.0153). ***D*1**, AEP response to the first (left) and second (right) standard sound of in control (black) versus *Tg(Nkx2.1-Cre);Syngap1^llox/+^* (red) mice. ***D*2**, The bar plot shows logarithmic ratio S2/S1 (*t* test; *t*_(11)_ = −2.5069, *p* = 0.0291). ***E*1**, ***E*2**, MMN pattern. ***E*1**, Superposed MMN (deviant–standard) traces. ***E*2**, Bar plots of MMN N1 (Wilcoxon rank sum test; *z* = −0.5123, *p* = 0.6084) and MMN P1 (Wilcoxon rank sum test; *z* = −1.0979, *p* = 0.2723) peak amplitude. Number of mice, *n* = 5 Ctrl; *n* = 8 Nkx2.1Cre-cHET, except for ***C*1*–C*3**, which is *n* = 5 Ctrl; *n* = 5 Nkx2.1Cre-cHET. Bar graphs represent mean ± SEM. *p < 0.05 and ***p < 0.001.

Neuronal synchronization is a core feature of cortical activity. In particular, neural oscillations in the gamma band (35–100 Hz) are associated with sensory processing and cognition (Fries, 2009; Uhlhaas and Singer, 2010; Gandal et al., 2012; Cardin, 2016). Of note, altered neural oscillations in the gamma range are thought to be a hallmark of neurodevelopmental disorders (Spencer et al., 2008; Peiker et al., 2015; Levin et al., 2017; Grent-'t-Jong et al., 2018; Gabard-Durnam et al., 2019; Carreno-Munoz et al., 2022). To assess the effects of *Syngap1* haploinsufficiency in Nkx2.1-expressing cells on cortical activity, we performed an extracellular recording of local field potentials (LFP) in cortical layer 5 of the auditory cortex of anesthetized adult mutant and control mice. Power density spectral analysis revealed significantly increased power in gamma frequency bands (30–100 Hz) in Nkx2.1-Cre cHET mice when compared with Nkx2.1-Cre controls (Fig. 3*A*). Recordings in awake mice, which were free to explore in an open field, showed similar results (Fig. 4*A*). Interestingly, we previously found increased baseline gamma power across different cortical regions of *Syngap1^+/−^* mice and *SYNGAP1* patients (Carreno-Munoz et al., 2022), suggesting that *Syngap1* haploinsufficiency restricted to Nkx2.1-expressing cells is sufficient to recapitulate abnormal cortical oscillation patterns caused by germline *Syngap1*/*SYNGAP1* haploinsufficiency.

### *Syngap1* haploinsufficiency in Nkx2.1-expressing interneurons leads to altered auditory cortex activity

We have previously observed that *Syngap1* haploinsufficient mice show alterations in specific aspects of auditory cortex activity, which were conserved in children carrying *SYNGAP1* mutations (Carreno-Munoz et al., 2022). Here, we investigated whether and how *Syngap1* haploinsufficiency in Nkx2.1-derived interneurons affects auditory cortex activity. Evoked potentials induced by a simple sensory stimulus, and described by EEG waveform activity within the first 500 ms after stimulus presentation, are the cortical responses most commonly used to study sensory perception in both humans and mouse models (Modi and Sahin, 2017). Analysis of auditory-evoked potentials (AEP) in Nkx2.1-Cre cHET and control littermates revealed no major difference in AEP amplitude and temporal dynamics between the two genotypes (Fig. 3*B*1*,B*2).

Rhythmic sensory stimulations oscillating at a certain frequency cause neural networks to oscillate in time to the stimulus, thus increasing the signal-to-noise ratio for activity at that frequency in the local cortical network, a process referred to as entrainment. Abnormal auditory entrainment following auditory stimulations has been reported in several neurodevelopmental syndromes, including fragile X syndrome and *Syngap1* haploinsufficiency (Ethridge et al., 2017; Jonak et al., 2020; Carreno-Munoz et al., 2022). We therefore recorded EEGs following a 40 Hz auditory stimulation and found significantly increased intertrial coherence in Nkx2.1-Cre cHET mice when compared with control littermates (Fig. 3*C*1–3), which is consistent with what was observed in *Syngap1* heterozygous mice (Carreno-Munoz et al., 2022).

Habituation responses to repeating tones (auditory gating), also called stimulus-specific adaptation, and novelty detection responses are frequently used as biomarkers in neurodevelopmental disorders and for the evaluation of treatment efficacy in ongoing clinical trials (Razak, Binder and Ethell, 2021). We investigated whether Nkx2.1-Cre cHET mice show deficits in stimulus-specific adaptation, by analyzing the cortical responses to 5–9 consecutive sounds presented at a fixed repetition rate of 1 Hz (Fig. 3*D*). We measured the N1 amplitude of AEP evoked by the first (S1) and second (S2) sound and calculated the ration between the two. Control littermates showed reduced S2 compared with S1 amplitude, while mutant mice showed no change (Fig. 3*D*), indicating a lack of stimulus-specific adaptation, again similar to what was observed in both *Syngap1^+/−^* mice and *SYNGAP1* haploinsufficient children (Carreno-Munoz et al., 2022).

Novelty detection response, also referred to as mismatch negativity (MMN), is a memory-based brain response to any discriminable change in a stream of auditory stimulation. Since MMN could not be observed in anesthetized wild-type mice, we performed this experiment in chronically implanted awake mice, which were free to explore in an open field (Fig. 4). Awake Nkx2.1-Cre cHET mice showed the same alterations in baseline gamma power, entrainment, and stimulus-specific adaptation we observed in anesthetized mice (Fig. 4*A–D*). To analyze MMN, we used a sequence of repetitive, standard sensory stimuli, interrupted by an oddball or deviant stimulus. In contrast to what was observed in *Syngap1^+/−^* mice (Carreno-Munoz et al., 2022), neither the mean amplitude of the first negative peak of MMN (N1), which indicates the sound-specific responses to the auditory stimuli, nor the expected positive peak at 70–110 ms after the stimulus presentation (P1) was affected in the Nkx2.1-Cre cHET mice, indicating that the deviant sound was detected correctly (Fig. 4*E*).

Altogether, these data suggest that *Syngap1* haploinsufficiency restricted to Nkx2.1-expressing neurons leads to altered auditory cortex activity, consistent with findings in both germline *Syngap1*^+/−^ mice and in *SYNGAP1* individuals.

### *Syngap1* haploinsufficiency restricted to postnatal PV interneurons leads to reduced stimulus-specific adaptation

**Figure 5.**
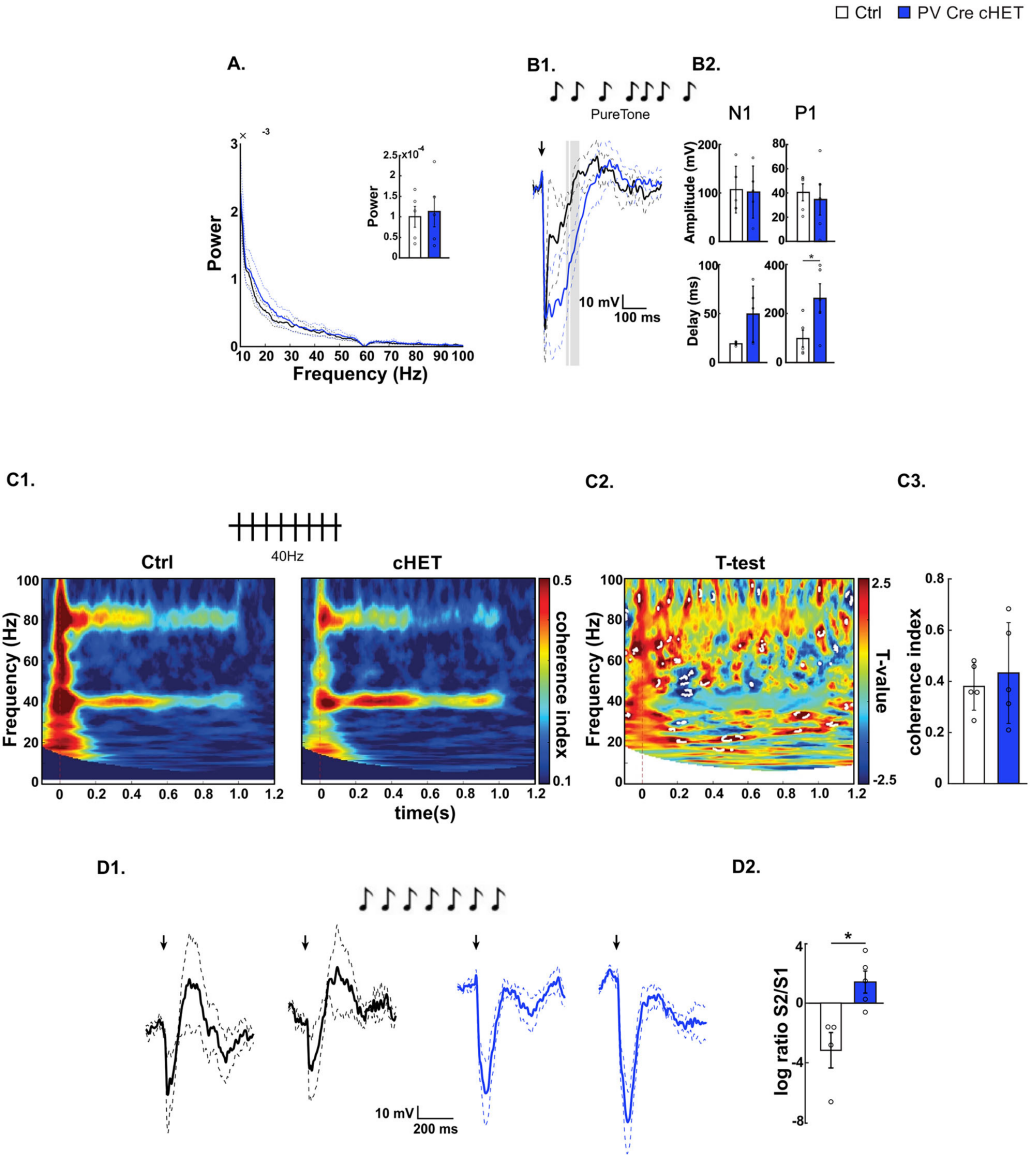
Postnatal-onset PV-specific *Syngap1* haploinsufficiency leads to decreased auditory habituation. ***A***, Power density spectra of 3 min baseline state (nonstimulus) EEG signal. The appended bar plot shows mean powers ± SEM of the broadband gamma (30–100 Hz) oscillations (*t* test; *t*_(8)_ = −0.2762, *p* = 0.7894). ***B*1**, ***B*2**, AEP profile. ***B*1**, Superposed grand average traces. The black arrow represents the beginning of the stimulation. Gray represents significant differences. ***B*2**, Bar plots showing N1 (left) amplitude (top panel, Wilcoxon rank sum test; *z* = 0, *p* = 1) and delay (bottom panel, Wilcoxon rank sum test; *z* = −1.6814, *p* = 0.0927) and P1 (right) amplitude (top panel, Wilcoxon rank sum test; *z* = 0.6267, *p* = 0.5309) and delay (bottom panel, *t* test; *t*_(8)_ = −2.3648, *p* = 0.0456). ***C*1–*C*3**, Auditory entrainment at 40 Hz. ***C*1**, Intertrial coherence in control (left) and Tg(PV-Cre);Syngap1^lox/+^ (right). ***C*2**, *t* test maps. Statistical differences (*p* < 0.05) are marked by white dotted lines. ***C*3**, Bar plots show coherence index values corresponding to the stimulating frequency, 40 Hz (Wilcoxon rank sum test; *z* = −0.2089, *p* = 0.8345). ***D*1**, AEP response to the first (left) and second (right) standard sound of control (black) versus Tg(PV-Cre);Syngap1^lox/+^ (red) mice. ***D*2**, Bar plot shows logarithmic ratio S2/S1 (*t* test; *t*_(7)_ = −3.4164, *p* = 0.0112). Number of mice, *n* = 5 Ctrl; *n* = 5 PVCre-cHET, except for ***D*1**, ***D*2**, that is, *n* = 4 Ctrl; *n* = 5 PVCre-cHET. Bar graphs represent mean ± SEM. *p < 0.05.

So far, our data show that Nkx2.1 cell–restricted prenatal *Syngap1* haploinsufficiency leads to alterations in gamma oscillation and selected aspects of auditory cortex activity. Among the different GABAergic cell populations, PV cell activity has been shown to modulate both gamma oscillations (Cardin et al., 2009; Sohal et al., 2009) and auditory habituation (Natan et al., 2015). Since the PV promoter only becomes active in the second postnatal week in cortical interneurons (del Rio et al., 1994), PV-Cre transgenic mice can be used to induce *Syngap1* haploinsufficiency postnatally in PV interneurons. EEG recordings showed no difference in baseline gamma oscillations between *PVCre;Syngap1^lox/+^* (PV-Cre cHET) and *PVCre;Syngap1^+/+^* (control) littermates (Fig. 5*A*). Auditory-evoked potential analysis, however, revealed that the P1 component, which is thought to represent inhibitory responses to auditory stimulation, was delayed in PV-Cre cHET mice (Fig. 5*B*1,2). Auditory entrainment following a 40 Hz auditory stimulation did not show any significant differences between the genotypes (Fig. 5*C*1–3). Conversely, stimulus-specific adaptation was absent in PV-Cre cHET mice (Fig. 5*D*), similar to what was observed in both Nkx2.1-Cre cHET (Fig. 2*D*), germline *Syngap1* heterozygous mice, and *SYNGAP1* haploinsufficient children (Carreno-Munoz et al., 2022). Altogether, these data suggest *Syngap1* expression in postnatal PV cells may still play a role in selective aspects of their function.

### *Syngap1* haploinsufficiency in SST interneurons does not affect auditory cortex activity

**Figure 6.**
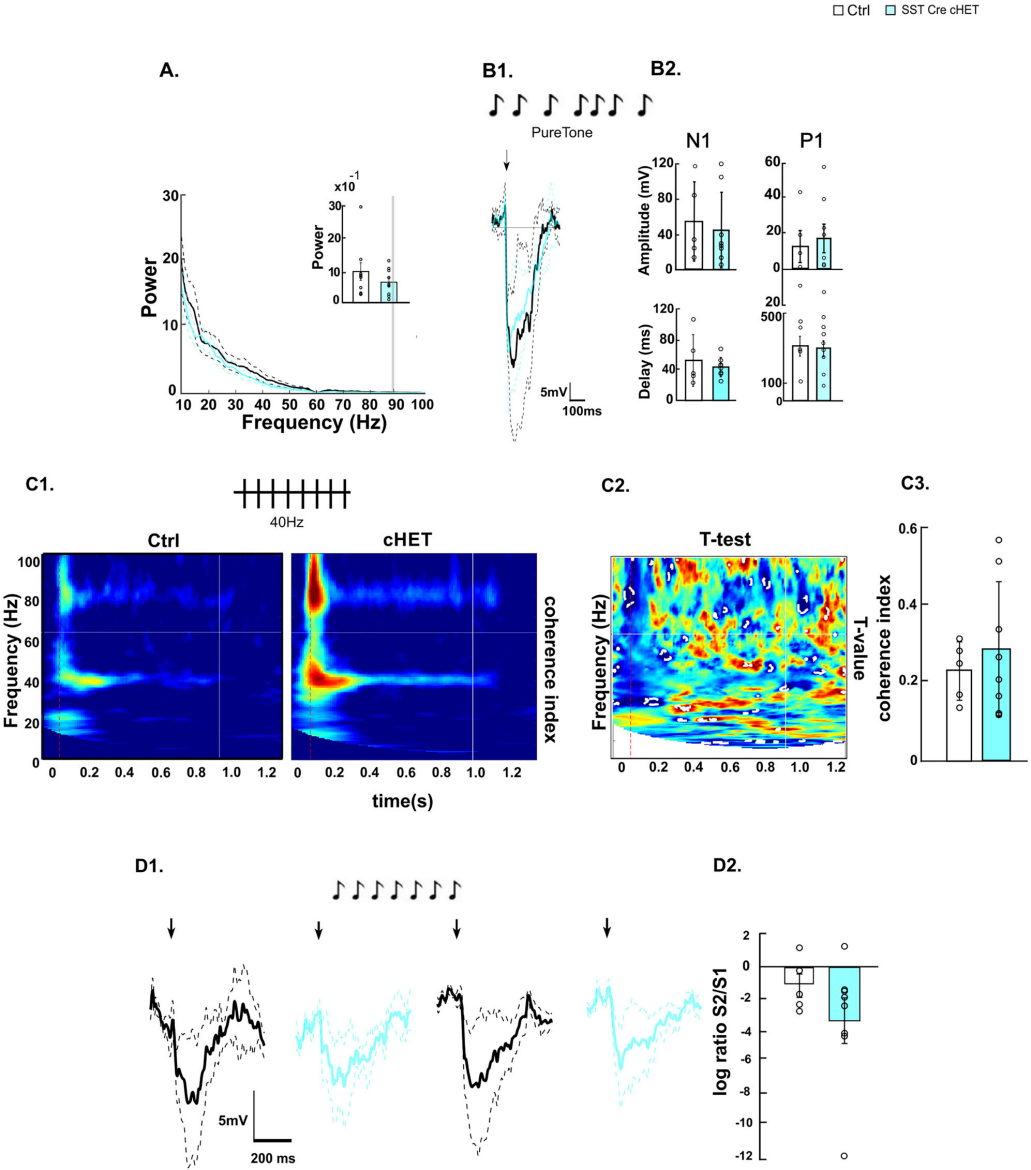
Prenatal-onset SST-specific *Syngap1* haploinsufficiency does not affect auditory stimulus processing. ***A***, Power density spectra of 3 min baseline state (nonstimulus) EEG signal. The appended bar plot shows mean powers ± SEM of the broadband gamma (30–100 Hz) oscillations (Wilcoxon rank sum; *z* = 0.7947, *p* = 0.4268). ***B*1**, ***B*2**. AEP profile. ***B*1**, Superposed grand average traces. The black arrow represents the beginning of the stimulation. ***B*2**, Bar plots showing N1 (left) amplitude (top panel, *t* test; *t*_(10)_ = 0.8783, *p* = 0.4004) and delay (bottom panel, *t* test; *t*_(10)_ = 1.0614, *p* = 0.3135) and P1 (right) amplitude (top panel, *t* test; *t*_(10)_ = 0.4141, *p* = 0.6875) and delay (bottom panel, *t* test; *t*_(10)_ = −0.6569, *p* = 0.5261). ***C*1–*C*3**, Auditory entrainment at 40 Hz. ***C*1**, Intertrial coherence in control (left) and Tg(SST-Cre);Syngap1^lox/+^ (right). ***C*2**, *t* test maps. Statistical differences (*p* < 0.05) are marked by white dotted lines. ***C*****3**, Bar plots show coherence index values corresponding to the stimulating frequency, 40 Hz (Wilcoxon rank sum test; *z* = 0.0589, *p* = 0.9530). ***D*1**, AEP response to the first (left) and second (right) standard sound of control (black) versus Tg(SST-Cre);Syngap1^lox/+^ (red) mice. ***D*2**, Bar plot shows logarithmic ratio S2/S1 (Wilcoxon signed rank; *z* = −0.5241, *p* = 0.6002]. Number of mice in ***A***, *n* = 9 Ctrl; *n* = 9 SST-Cre-cHET; ***B*1**, ***B*2**, *n* = 5 Ctrl; *n* = 7 SST-Cre-cHET; ***B*3**, ***B*4**, *n* = 5 Ctrl; *n* = 9 SST-Cre-cHET; ***C*1*–C*3**, *n* = 6 Ctrl; *n* = 9 SST-Cre-cHET; ***D*1**, ***D*2**, *n* = 6 Ctrl; *n* = 6 SST-Cre-cHET. Bar graphs represent mean ± SEM. *p < 0.05.

Nkx2.1 drives Cre expression in both cortical PV and SST interneuron progenitors. In contrast to PV, which is expressed only postnatally, SST is highly expressed in developing cortical GABA neurons starting from midgestation (Batista-Brito et al., 2008). SST-Cre expression initiates prenatally, as early as E13, just after the neurons have started their tangential migration (Taniguchi et al., 2011); therefore, SST-Cre mice allow for prenatal manipulation of *Syngap1* in SST neurons. Power density spectrum analysis did not reveal any significant alteration in LFP activity in *SstCre;Syngap1^lox/+^* (Sst-Cre cHET) mice compared with *SstCre;Syngap^+/+^* control littermates (Fig. 6*A*). Furthermore, we could not detect any significant differences between the two genotypes in auditory-evoked potentials (Fig. 6*B*). Finally, neither auditory entrainment following a 40 Hz auditory stimulation (Fig. 6*C*) nor stimulus-specific adaptation (Fig. 6*D*) revealed any significant difference between Sst-Cre cHET and control littermates. Altogether, these data suggest that any potential alteration in SST interneuron function does not significantly contribute to the auditory cortex activity alterations observed in Nkx2.1-Cre cHET mice.

### Mice with *Syngap1* haploinsufficiency restricted to Nkx2.1-expressing interneurons show deficits in social behavior and extinction of fear memory

**Figure 7.**
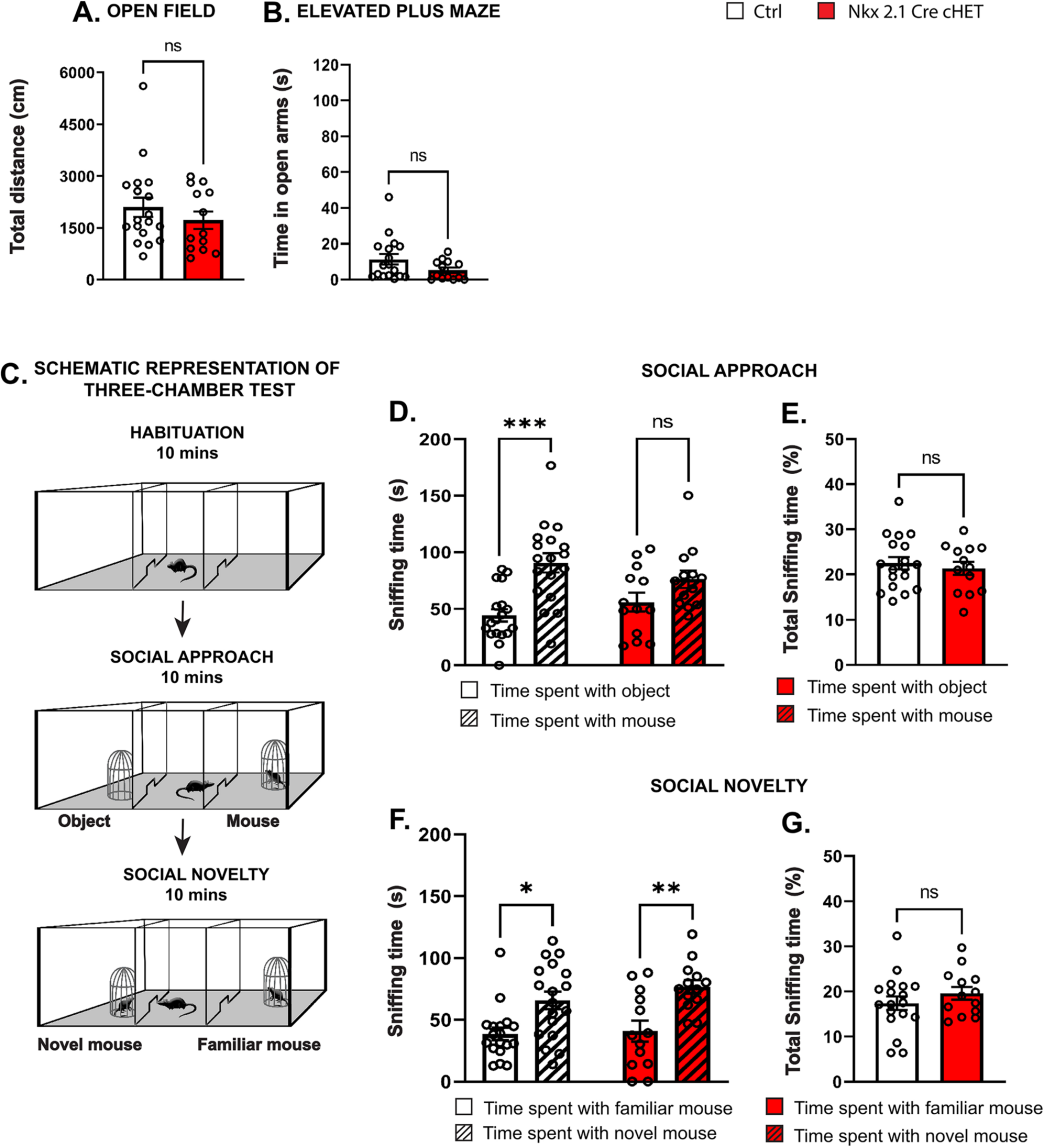
MGE-specific *Syngap1* haploinsufficient mice show social behavior deficits. ***A***, Distance traveled in the open field (Mann–Whitney test *p* = 0.4175). Number of mice, *n* = 18 Ctrl; *n* = 13 Nkx2.1-Cre cHET. ***B***, Time spent in open arms of the elevated plus maze (Mann–Whitney test *p* = 0.0714). Number of mice, *n* = 17 Ctrl; *n* = 13 Nkx2.1Cre-cHET. ***C***, Schematic representation of the three-chamber test. ***D***, Social approach test. Ctrl mice spend significantly more time sniffing the mouse over the object, while and Nkx2.1Cre-cHET do not (two-way ANOVA, *F*_(1,29)_ genotype = 0.04618 *p* = 0.8313, *F*_(1,29)_ time = 13.80 *p = 0.0009, *F*_(1,29)_ genotype*time = 2.126 *p* = 0.1555; Sidak's multiple-comparisons post hoc test for time spent with object vs mouse; Ctrl *p* = 0.0008, Nkx2.1Cre-cHET *p* = 0.2765). ***E***, Total sniffing time in social approach test (Welch's *t* test *p* = 0.5822). ***F***, Social novelty test. Both Ctrl and Nkx2.1Cre-cHET spent more time sniffing the novel mouse over the familiar one (two-way ANOVA, *F*_(1,29)_ genotype = 1.093 *p* = 0.3045, *F*_(1,29)_ time = 19.97 *p = 0.0001, *F*_(1,29)_ genotype*time = 0.3715 *p* = 0.5469; Sidak's multiple-comparisons post hoc test for time spent with familiar mouse vs novel mouse; Ctrl *p* = 0.0115, Nkx2.1Cre-cHET *p* = 0.0047). ***G***, Total sniffing time in Social Novelty test (Welch's *t* test *p* = 0.287). ***D–G***, Number of mice, *n* = 18 Ctrl; *n* = 13 Nkx2.1Cre-cHET. Bar graphs represent mean ± SEM. *p* > 0.05, not significant (ns), *p < 0.05, **p < 0.01, ***p < 0.001.

**Figure 8.**
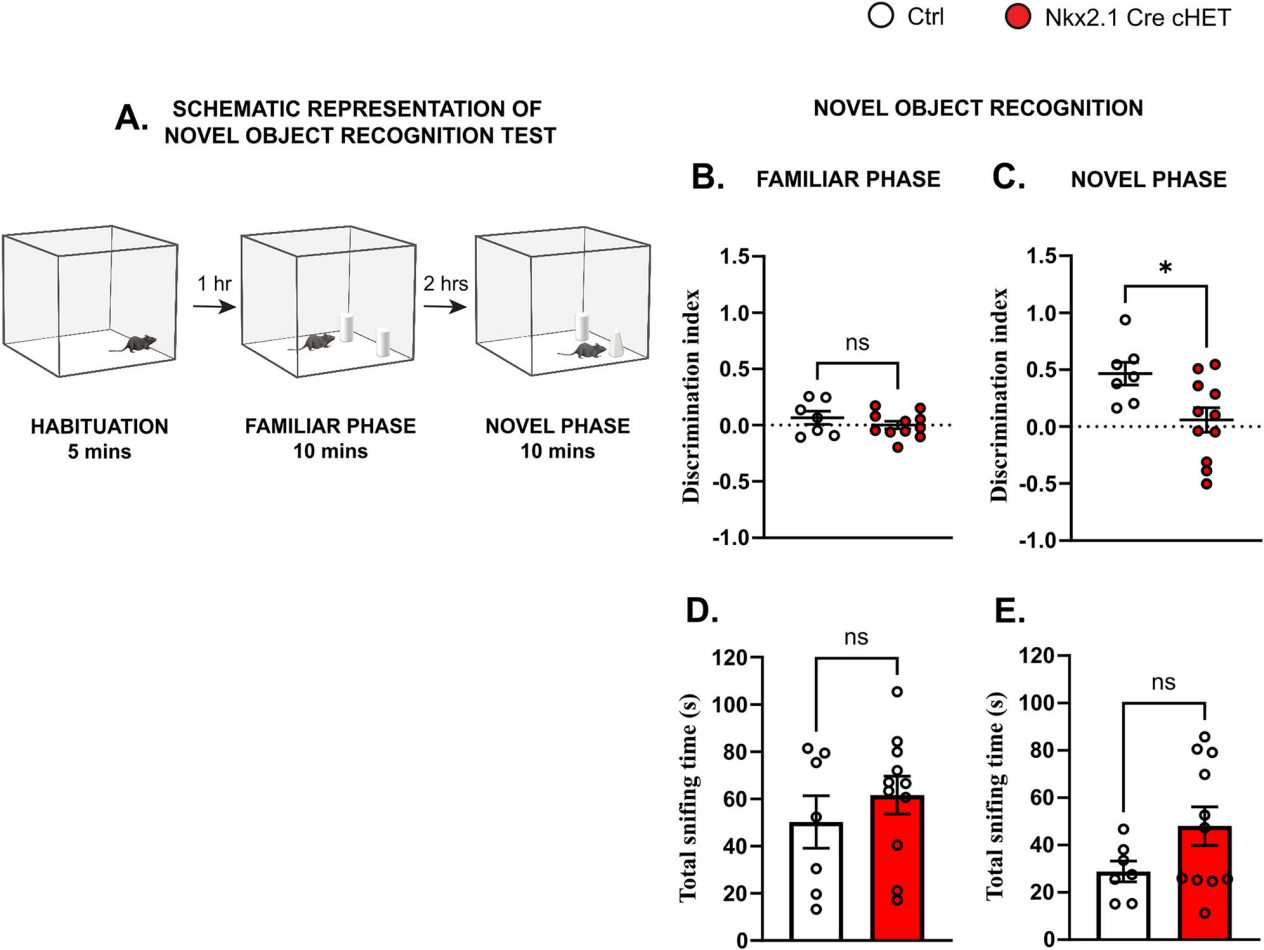
MGE-restricted *Syngap1* haploinsufficient mice have memory deficits in novel object recognition. ***A***, Schematic representation of novel object recognition test. ***B***, ***D***, Discrimination index (top, Mann–Whitney test *p* = 0.5360) and total sniffing time (bottom, Mann–Whitney test *p* = 0.5360) in the familiar phase. ***C***, ***E***, Discrimination index (top, Mann–Whitney test *p* = 0.0204) and total sniffing time (bottom, Mann–Whitney test *p* = 0.2463) in novel object phase. Number of mice, *n* = 7 Ctrl and *n* = 11 Nkx2.1-Cre cHET. ns, not significant, *p < 0.05.

Different *Syngap1* mouse models consistently showed behavioral and cognitive problems in specific domains, including hyperactivity in the open field and increased time spent in the open arm of the elevated plus maze, which may indicate reduced innate fear leading to an increase in risk-taking (Kilinc et al., 2018), deficits in spatial learning, remote contextual fear memory consolidation and weaker social memory (Komiyama et al., 2002; Guo et al., 2009; Muhia et al., 2010; Clement et al., 2012; Ozkan et al., 2014; Berryer et al., 2016; Kilinc et al., 2022). We sought to investigate whether *Syngap1* haploinsufficiency specifically in Nkx2.1-expressing neurons is sufficient to lead to the development of cognitive and behavioral alterations. We did not find any significant differences in the distance covered in an open field (Fig. 7*A*) or in the time spent in the open arms of the elevated plus maze (Fig. 7*B*) between Nkx2.1-Cre cHET and control littermates, in contrast to *Syngap1^+/−^* mice which displayed hyperactivity and reduced anxiety/higher risk-taking (Clement et al., 2012; Berryer et al., 2016). Conversely, in the three-chamber test (Fig. 7*C–G*), we observed that while control mice spent more time interacting with a mouse compared with exploring an object, Nkx2.1-Cre cHET mice spent equal time with both, suggesting significantly weaker social preference behavior as compared with controls (Fig. 7*D*). This was not due to altered exploratory behavior, since total sniffing time did not differ between genotypes (Fig. 7*E*). Of note, both mutant and control mice preferred sniffing a novel versus a familiar mouse when this was introduced immediately after the social approach testing phase (Fig. 7*F*), indicating intact social recognition memory. In addition, Nkx2.1-Cre cHET mice failed to show preference toward a novel object when it was presented 2 h after the familiar phase (two identical objects in the open field), in the novel recognition test (Fig. 8). It is thus possible that recognition memory impairments appear when the memory task becomes more challenging. Alternatively, different memory tasks may involve distinct brain regions differently affected by *Syngap1* conditional haploinsufficiency.

**Figure 9.**
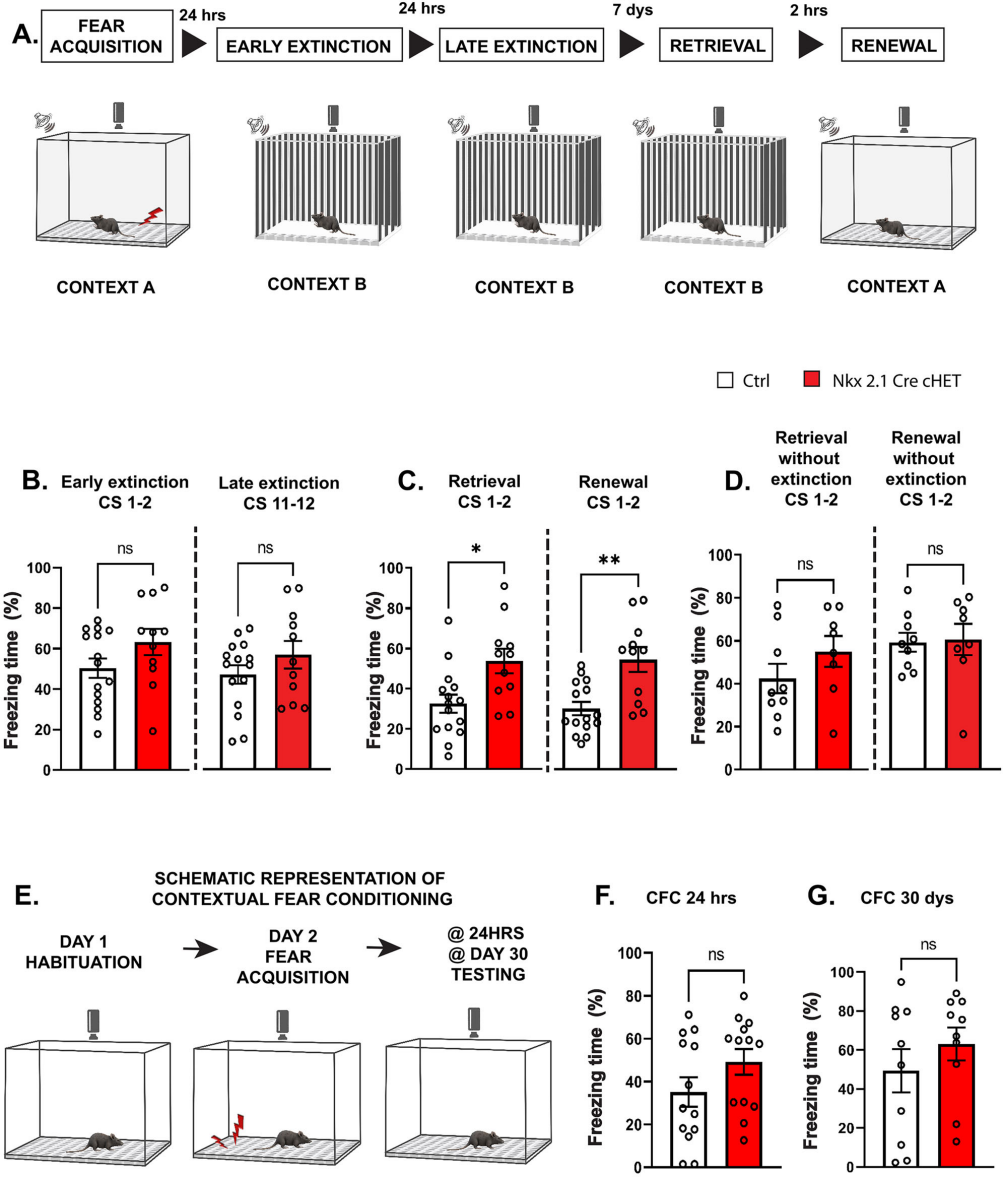
MGE-specific *Syngap1* haploinsufficient mice are unable to extinguish cue-associated fear memories. ***A***, Schematic representation of fear extinction protocol. ***B***, Percentage of freezing during first two CS (Welch's *t* test *p* = 0.1227) and last two CS (Welch's *t* test *p* = 0.2497) of the extinction training. ***C***, Nkx2.1Cre-cHET mice show a higher percentage of freezing during retrieval (Welch's *t* test, *p* = 0.0113) and renewal (Welch's *t* test, *p* = 0.0032) test compared with Ctrl littermates. ***B***, ***C***, Number of mice, *n* = 15 Ctrl mice; *n* = 11 Nkx2.1Cre-cHET. ***D***, Freezing time 10 d after fear memory acquisition in the absence of extinction training (retrieval, Welch's *t* test *p* = 0.2236; renewal, Welch's *t* test *p* = 0.8780; ns, not significant). Number of mice, *n* = 9 Ctrl; *n* = 8 Nkx2.1Cre-cHET. ***E***, Schematic representation of contextual fear conditioning protocol. ***F***, ***G***. Percentage of freezing 24 h (***F***, Welch's *t* test *p* = 0.1357) and 30 d (***G***, Welch's *t* test *p* = 0.3381) after fear acquisition. Number of mice, ***F***, *n* = 13 for both genotypes; ***G***, *n* = 10 for both genotypes. Bar graphs represent mean ± SEM. *p* > 0.05, not significant (ns), *p < 0.05, **p < 0.01, ***p < 0.001.

GABAergic cell activity modulates behaviors linked to cognitive flexibility, such as the extinction of a learned behavior (Sparta et al., 2014). Therefore, we next sought to understand whether cognitive flexibility was affected in the Nkx2.1-Cre cHET mice, by analyzing extinction of cue-mediated aversive behavior. In particular, we measured fear memory acquisition, extinction, and spontaneous recovery in Nkx2.1-Cre cHET mice as compared with control littermates (Fig. 9*A–D*). During cue (sound)-mediated fear conditioning, both Nkx2.1-Cre cHET and control mice presented strong freezing behavior (repeated measure ANOVA with Sidak's multiple-comparisons test; CS-US1 *p* = 0.8732, CS-US2 *p* = 0.7456, CS-US3 *p* = 0.7096, CS-US4 *p* = 0.8705, CS-US5 *p* = 0.9998; number of mice, *n* = 15 Ctrl and *n* = 11 Nkx2.1-Cre cHET), suggesting that fear learning was not affected by *Syngap1* haploinsufficiency in Nkx2.1-expressing neurons. On the first and second day of extinction training (early extinction, CS 1–2, and late extinction, CS 11–12), mutant mice showed freezing times similar to control littermates (Fig. 9*B*). However, significant differences became apparent during both retrieval and renewal tests 7 d later (Fig. 9*C*), suggesting a lack of extinction learning consolidation and stronger recovery of fear memory in Nkx2.1-Cre cHET mice. The increased fear expression during retrieval and renewal was not due to the formation of stronger fear memories, since in the absence of extinction training, freezing levels were indistinguishable between conditional heterozygous and control mice 10 d after fear conditioning (Fig. 9*D*). Furthermore, Nkx2.1-Cre cHET mice did not show increased freezing while testing for contextual fear memory 24 h or 30 d after fear acquisition (Fig. 9*E–G*), indicating normal consolidation of remote contextual fear memories. Finally, the significant increase in freezing behavior observed in Nkx2.1-Cre cHET while testing for fear retrieval, 7 d after the extinction protocol, was not due to altered motor or anxiety behavior, since we found no difference between the genotypes in both the open field and elevated plus maze assays (Fig. 7*A,B*).

Overall, our data suggest that Nkx2.1 cell–restricted *Syngap1* haploinsufficiency leads to specific deficits in social behavior (preference of a mouse over an inanimate object) and fear extinction learning.

### *Syngap1* haploinsufficiency restricted to either PV or SST interneurons does not cause deficits in cognitive flexibility or social behavior

**Figure 10.**
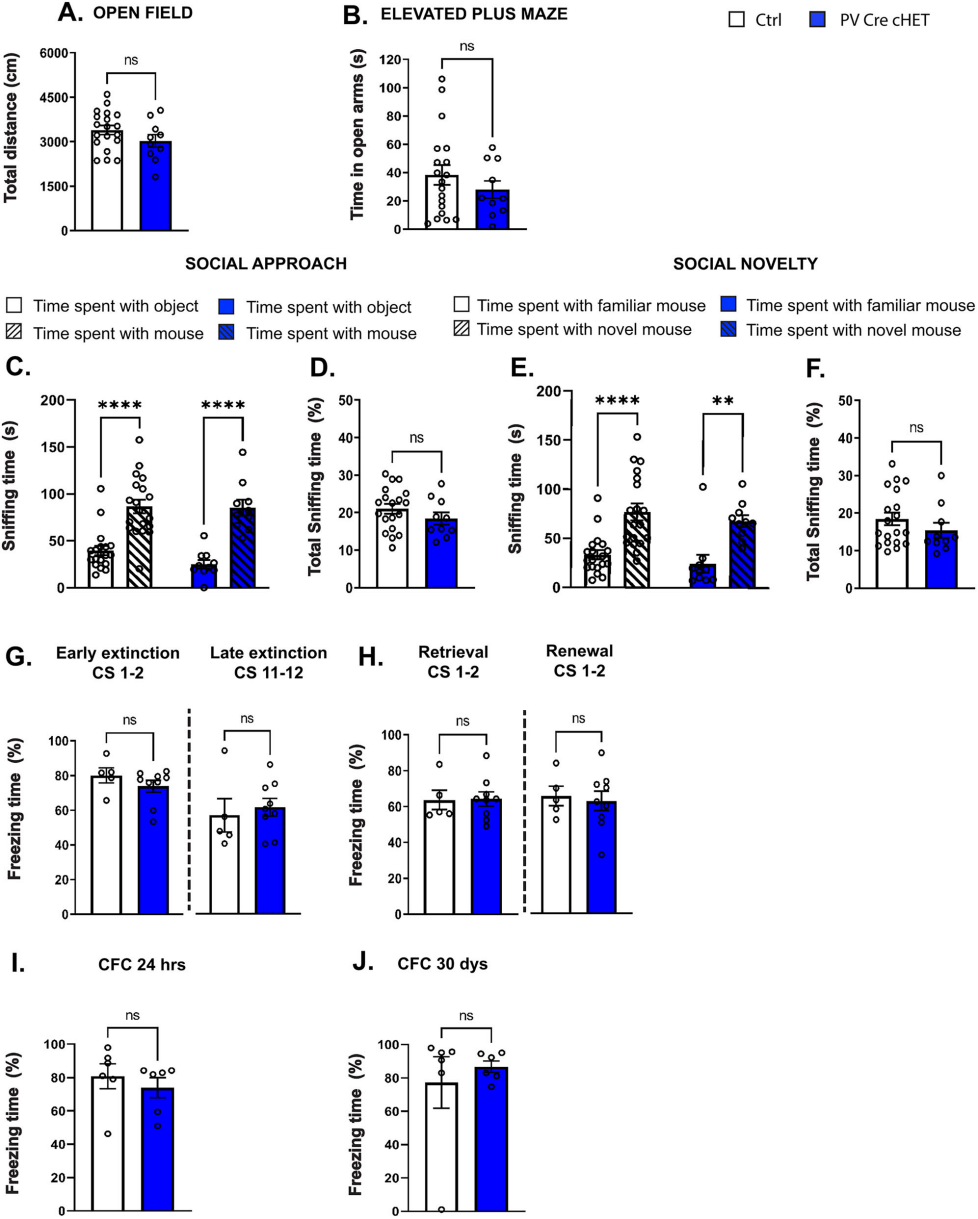
PV-specific *Syngap1* haploinsufficient mice do not show social or cognitive flexibility deficits. ***A***, Total distance traveled in the open field test (Welch's *t* test, *p* = 0.1782). ***B***, Time spent in the open arms of the elevated plus maze (Welch's *t* test, *p* = 0.2735). ***C***, Social approach (two-way ANOVA, *F*_(1,27)_ genotype = 1.432 *p* = 0.2419, *F*_(1,27)_ time = 50.38 *p ≤ 0.0001, *F*_(1,27)_ genotype*time = 0.7512 *p* = 0.3937; Sidak's multiple-comparisons post hoc test for time spent with object vs mouse; Ctrl *p* ≤ 0.0001, PVCre-cHET *p* ≤ 0.0001). ***D***, Total sniffing time in social approach test (Welch's *t* test, *p* = 0.2295). ***E***, Social novelty (two-way ANOVA, *F*_(1,27)_ genotype = 1.185 *p* = 0.2860, *F*_(1,27)_ time = 35.79 *p ≤ 0.0001, *F*_(1,27)_ genotype*time = 3.659 × 10^−6^ *p* = 0.9985; Sidak's multiple-comparisons post hoc test for time spent time with familiar mouse vs novel mouse; Ctrl *p* ≤ 0.0001, PVCre-cHET *p* = 0.0020). ***F***, Total sniffing time in social novelty (Mann–Whitney test, *p* = 0.3077). ***A–F***, Number of mice, *n* = 19 Ctrl; *n* = 10 PVCre-cHET. ***G***, Percentage of freezing during first two (Mann–Whitney test *p* = 0.2398) and last two CS (Welch's *t* test *p* = 0.6828) of extinction training. ***H***, Percentage of freezing during the retrieval (Welch's *t* test *p* = 0.9314) and renewal test (Welch's *t* test *p* = 0.7213). ***G***, ***H***, Number of mice, *n* = 5 Ctrl, *n* = 9 PVCre-cHET. ***I***, ***J***, Percentage of freezing 24 h (***I***, Mann–Whitney test *p* = 0.5887) and 30 d (***J***, Mann–Whitney test *p* = 0.3939) after contextual fear acquisition. ***I***, ***J***, Number of mice, *n* = 6 for both genotypes. Bar graphs represent mean ± SEM. *p* > 0.05, not significant (ns), *p < 0.05, **p < 0.01, ***p < 0.001, ****p < 0.0001.

**Figure 11.**
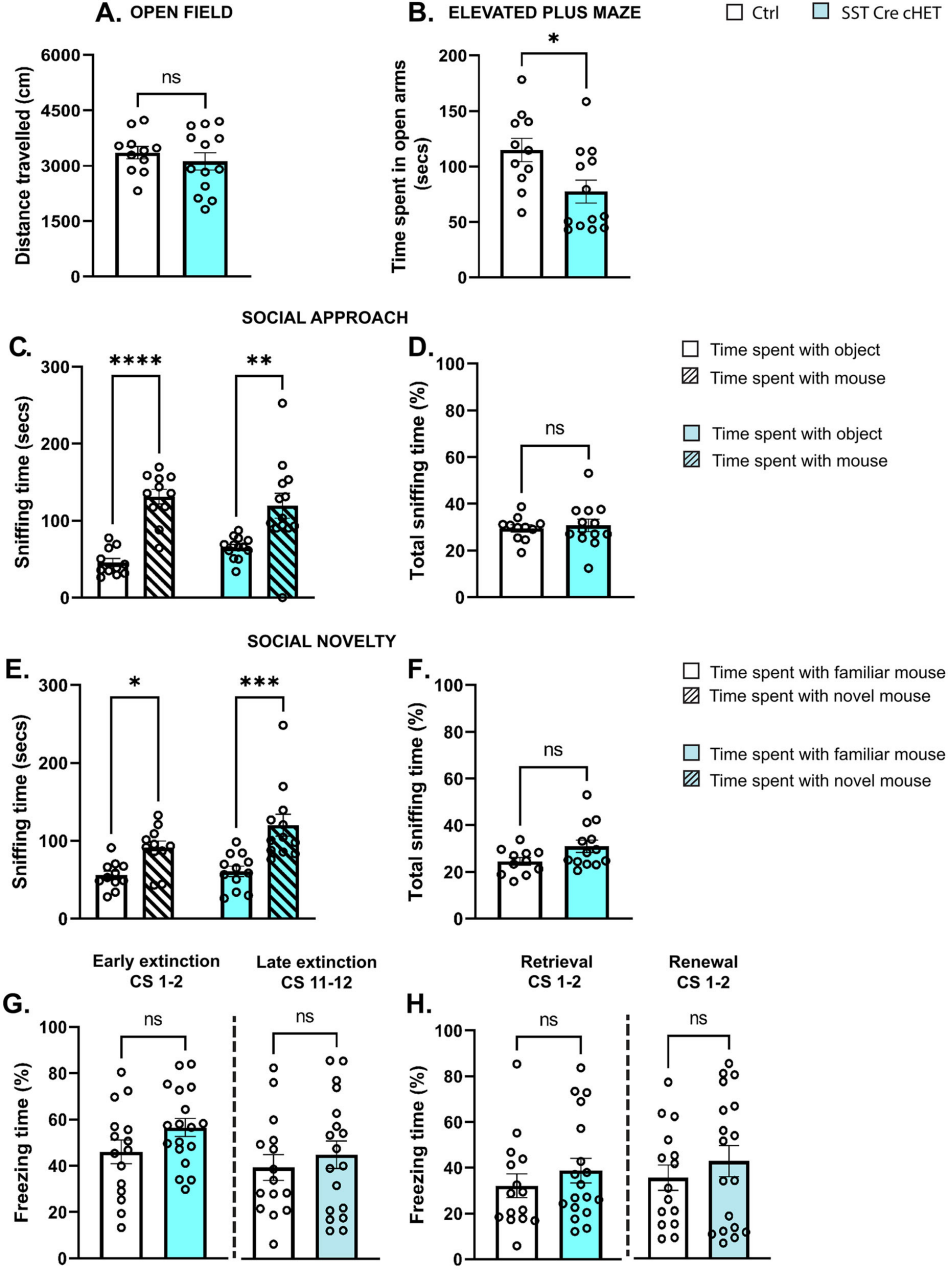
SST-specific *Syngap1* haploinsufficient mice do not show social or cognitive flexibility deficits. ***A***, Total distance traveled in the open field test (Welch's *t* test, *p* = 0.4340). ***B***, Time spent in the open arms of the elevated plus maze (Welch's *t* test, *p = 0.0185). ***C***, Social approach (two-way ANOVA, *F*_(1,22)_ genotype = 0.1586 *p* = 0.6943, *F*_(1,22)_ time = 39.92 *p ≤ 0.0001, *F*_(1,22)_ genotype*time = 1.964 *p* = 0.1750; Sidak's multiple-comparisons post hoc test for time spent with object vs mouse; Ctrl *p ≤ 0.0001, PVCre-cHET *p = 0.0029). ***D***, Total sniffing time in social approach test (Welch's *t* test, *p* = 0.6808). ***E***, Social novelty (two-way ANOVA, *F*_(1,21)_ genotype = 3.0 *p* = 0.0979, *F*_(1,22)_ time = 27.67 *p ≤ 0.0001, *F*_(1,22)_ genotype*time = 1.720 *p* = 0.2039; Sidak's multiple-comparisons post hoc test for time spent time with familiar mouse vs novel mouse; Ctrl *p = 0.0247, PVCre-cHET *p = 0.0002). ***F***, Total sniffing time in social novelty (Welch's *t* test, *p* = 0.0507). ***A–F***, Number of mice, *n* = 11 Ctrl; *n* = 13 PVCre-cHET. ***G***, Percentage of freezing during first two (Welch's *t* test *p* = 0.1105) and last two CS (Welch's *t* test *p* = 0.5027) of extinction training. ***H***, Percentage of freezing during the retrieval (Mann–Whitney test *p* = 0.4421) and renewal test (Mann–Whitney test *p* = 0.5320). ***G***, ***H***, Number of mice, *n* = 15 Ctrl, *n* = 18 PVCre-cHET. Bar graphs represent mean ± SEM. *p* > 0.05, not significant (ns), *p < 0.05, **p < 0.01, ***p < 0.001, ****p < 0.0001.

Finally, we investigated whether postnatal onset of *Syngap1* haploinsufficiency restricted to PV cells affected mouse behavior and cognition, and found no difference in motor activity (Fig. 10*A*), anxiety (Fig. 10*B*), social behavior (Fig. 10*C–F*), cued and contextual short-term fear memory (Fig. 10*G,I*), extinction of cue-based fear memories (Fig. 10*G,H*), or consolidation of remote contextual fear memory (Fig. 10*J*). We then asked whether *Syngap1* haploinsufficiency in SST-expressing neurons may affect mouse behavior. We found that Sst-Cre cHET mice spent less time in the open arms of the elevated plus maze compared with their littermates (Fig. 11*B*), suggesting increased anxiety, but no deficits in any of the other tested behaviors (Fig. 11*A,C–H*).

## Discussion

While several studies suggest a role of *Syngap1* in GABAergic interneurons, whether and when distinct GABAergic interneurons populations are more sensitive to *Syngap1* haploinsufficiency were open questions. The major finding of this study is that prenatal-onset *Syngap1* haploinsufficiency restricted to Nkx2.1-expressing GABAergic interneuron precursors leads to the development of altered auditory cortex activity, impaired sociability, and cognitive inflexibility, indicated by the inability to extinguish fear memories. Prenatal-onset *Syngap1* haploinsufficiency restricted to SST interneurons, one of the two major classes of cortical GABAergic interneurons derived from Nkx2.1-expressing precursors, does not recapitulate any of the observed phenotypes, whereas postnatal-onset *Syngap1* haploinsufficiency restricted to PV interneurons, the other major cortical GABAergic population derived from the MGE, leads to absence of stimulus-specific adaptation in the auditory cortex, but no other behavioral phenotypes.

One possible explanation of these results is that early and pre- or perinatal, but not postnatal, *Syngap1* haploinsufficiency in PV cells may have a significant impact on the development of social behavior and ability to extinguish fear memories in adulthood and on the aspects of auditory cortex activity, such as gamma oscillation power and auditory entrainment, which are altered in Nkx2.1-Cre cHet but not in PV-Cre cHet mice. To test this hypothesis, it will be desirable to induce *Syngap1* haploinsufficiency in putative PV cells starting at the embryonic postmitotic stage. Unfortunately, the expression onset of the known markers specific to these interneuron populations, such as PV itself or synaptotagmin 2 (J. Xu, Mashimo and Sudhof, 2007; Sommeijer and Levelt, 2012; Miyamae et al., 2017; Ramaswamy, 2017), is postnatal; therefore, this strategy is currently not feasible.

Another, not mutually exclusive hypothesis, is that *Syngap1* haploinsufficiency in neurons other than GABAergic cells may contribute to the phenotypes observed in Nkx2.1-Cre cHet mice. In fact, Nkx2.1 is expressed in specific cholinergic neurons (Magno et al., 2017); however, whether *Syngap1* is expressed by these neurons, and, if this is the case, to what extent haploinsufficiency of *Syngap1* in cholinergic cells in different brain regions, such as the striatum and basal forebrain, may affect social behavior and cognitive flexibility remains to be established.

Supporting the hypothesis that *Syngap1* haploinsufficiency may affect PV interneuron function, we have recently discovered that *Syngap1* haploinsufficiency restricted to Nkx2.1-expressing interneurons reduces PV but not SST interneuron excitability in the primary auditory cortex, possibly by a mechanism dependent on D-type K^+^ currents (Francavilla et al., 2024). Of note, PV cell intrinsic properties mature during early postnatal development, at a time window where *Syngap1* expression may still be comparable to control levels in PV-Cre cHet mice, due to the later timing of PV expression onset. In addition to reduced excitability, PV cells in Nkx2.1-Cre cHet mice have reduced glutamatergic drive, suggesting that they may be hypoactive (Francavilla et al., 2024). In PV interneurons, connectivity and cell excitability are reciprocally regulated at the circuit level (Favuzzi et al., 2017); thus, we could speculate that early-onset *Syngap1* haploinsufficiency in MGE-derived interneurons may affect the development of their intrinsic excitability properties, which in turn would modulate the maturation of their excitatory drive. Alternatively, homeostatic adaptation of PV interneurons in response to the decreased number of excitatory inputs could trigger changes in Kv1 channels (Dehorter et al., 2015; Favuzzi et al., 2017). As a result of these developmental changes, altered PV cell function could initiate secondary alterations, which may impact network activity or/and local activity-dependent molecular signaling and thereby the developmental trajectory of other neurons, thus leading to the emergence of cognitive and behavioral alterations. Furthermore, Syngap1 has been shown to regulate interneuron migration as well (Su et al., 2019). We did not detect any difference in PV and SST cell distribution in adult mutant mice; however, we cannot exclude that the time course of PV cell migration might be altered, thus affecting neuron circuit assembly. Finally, based on the proportion of PV and SST neurons expressing GFP, a significant proportion of MGE-derived cortical interneurons likely did not undergo *Syngap1* recombination in Nkx1.2-Cre cHet mice, possibly leading to milder phenotypes than if all MGE-derived interneurons would be affected.

Alterations of adult PV interneuron function caused by haploinsufficiency of *Syngap1* during early development might contribute to the phenotypes observed in Nkx2.1-Cre cHet mice. For example, recent studies have linked deficits in PV interneuron–mediated inhibition to increased baseline cortical gamma rhythmic activity (Carlén et al., 2012; Chen et al., 2017; Guyon et al., 2021). PV cells have also been shown to amplify habituation, and optogenetic suppression of PV cells led to increased responses to both repetitive (standard) and rare (deviant) tones (Natan et al., 2015). Altered prefrontal PV interneuron activity could also contribute to the observed absence of fear extinction learning in Nkx2.1-Cre cHet mice. For example, two mouse models carrying PV cell–restricted deletion of a gene associated with reduced synaptic plasticity, the histone deacetylase 2 (Hdac2; Lavertu-Jolin et al., 2023) and the neuronal receptor for myelin-associated growth inhibitors, nogo receptor 1 (Bhagat et al., 2016), respectively, show increased remodeling of PV interneuron synapses and reduced spontaneous recovery of fear memory in adult mice following extinction training. In contrast, PV cell–specific deletion of the neurotrophin receptor p75 nudges prefrontal PV interneurons toward a less plastic state, limiting their recruitment to activating stimuli and impairing fear memory extinction learning (Chehrazi et al., 2023). PV interneurons have also been implicated in social behavior. Perturbing excitation/inhibition balance in the rodent medial prefrontal cortex using optogenetic tools led to social behavior deficits, which can be partially rescued by stimulation of PV neurons (Yizhar et al., 2011). In addition, increasing the excitability of prefrontal PV interneurons using optogenetic approaches rescued deficits in social behavior in mice lacking the autism-linked gene *CNTNAP2* (Selimbeyoglu et al., 2017). Impaired social behavior was observed in mouse models carrying Nkx2.1 or PV interneuron–specific haploinsufficiency of tuberous sclerosis 1 (*TSc1*; Amegandjin et al., 2021), which in humans causes tuberous sclerosis, a disorder associated with seizures, intellectual disability, and autism. To causally link PV cell dysfunction to the observed phenotypes, it will be necessary to test whether manipulation of PV cell activity by targeted chemogenetic or pharmacological approaches (as previously described in Kourdougli et al., 2023) at different developmental stages rescues any of the observed phenotypes.

Using a different Syngap1-lox line, we have previously shown that *Syngap1* haploinsufficiency in Nkx2.1-derived neurons leads to reduced perisomatic PV cell boutons during the first postnatal month, abnormal gamma oscillation power in the motor cortex during active exploration, and reduced sociability (Berryer et al., 2016). However, in these transgenic mice, we noticed reduced numbers of PV and SST neurons, possibly due to the presence of an inverted LoxP site (Khlaifia et al., 2023). Here, using a different Syngap1-lox line, we confirmed that *Syngap1* haploinsufficiency restricted to Nkx2.1-derived cells leads to social behavior dysfunction, in the absence of any significant difference in PV and SST interneurons density between conditional heterozygous and control littermates. In addition, we found altered extinction of fear memory, suggestive of impaired cognitive flexibility, and abnormal auditory cortex activity both at baseline and following complex auditory stimulations. Of note, these auditory phenotypes have been observed also in *Syngap1^+/−^* mice and SYNGAP1-ID patients (Carreno-Munoz et al., 2022), suggesting that *Syngap1* haploinsufficiency in MGE-derived cells may contribute to these phenotypes. Whether and how GABAergic inhibition, possibly mediated by different interneuron populations, is altered by *SYNGAP1* haploinsufficiency in the human brain is as yet unknown.

*Syngap1*/*SYNGAP1* haploinsufficiency is associated with epileptic encephalopathy in humans and altered ictal activity and threshold to seizure induction in mice (Clement et al., 2012; Berryer et al., 2013; Vlaskamp et al., 2019; Carreno-Munoz et al., 2022). Increased ictal activity affects several developmental processes which are known to be dependent on neuronal activity and experience. However, we did not detect any ictal activity in *Nkx2.1Cre;Syngap1^lox/+^* mice, contrary to what we observed in *Syngap1^+/−^* mice (Carreno-Munoz et al., 2022); thus, we can exclude circuit hyperexcitability as a confounding factor in the observed behavioral alterations.

Our data show that prenatal-onset haploinsufficiency of *Syngap1* in SST interneurons does not lead to either extinction learning and sociability deficits or cause altered auditory stimulus processing in adult mutant mice. We detected *Syngap1* mRNA in SST neurons; however, whether and to what extent the protein is produced remains to be established. Furthermore, *Syngap1* has different isoforms, which appear to play different roles in glutamatergic synapse plasticity and cognitive functions (McMahon et al., 2012; Araki et al., 2020; Gou et al., 2020); it is therefore possible that certain isoforms may selectively play distinct roles at different developmental stages and in different interneuron types. It is also possible that *Syngap1* haploinsufficiency in SST neurons might contribute to phenotypes other than those tested in this study, including altered anxiety (Fig. 8), which thus remains to be explored.

In summary, collectively our results, together with other findings (Francavilla et al., 2024), suggest that Syngap1 plays a role in PV interneuron development during a critical pre- or/and perinatal developmental window and adds to the growing evidence highlighting the contribution of cortical PV inhibitory interneurons to sensory and cognitive abnormalities in neurodevelopmental disorders.
